# Structural insights into metallocluster trafficking in the nitrogenase assembly scaffold NifEN

**DOI:** 10.1038/s41929-026-01489-9

**Published:** 2026-03-03

**Authors:** Bryan Neumann, Kristal A. Brandon, Robert Quechol, Diana S. Suder, Chi Chung Lee, Yimo Yang, Kamil Górecki, Jared A. Wiig, Yilin Hu, Shane Gonen, Markus W. Ribbe

**Affiliations:** 1 Department of Molecular Biology and Biochemistry, University of California, Irvine, CA USA; 2 Department of Chemistry, University of California, Irvine, CA USA; 3 Present Address: AbbVie, Irvine, CA USA

**Keywords:** Cryoelectron microscopy, Metalloproteins

## Abstract

Nitrogenase catalyses small-molecule activation, and has great relevance to agronomy, environment and energy. Understanding the assembly of the complex nitrogenase cofactor is a decades-long goal in the field, and structural insights into this process remain scarce. Here we report a cryogenic electron microscopy (cryo-EM) study of heterologously expressed NifEN, a key player converting the precursor (L-cluster) to a mature cofactor (M-cluster). Structural analyses of apo- and holo-NifEN demonstrate major conformational changes triggered by L-cluster incorporation. Further examinations of NifEN structures with inwardly and outwardly bound L-clusters, coupled with supporting mutational studies, AlphaFold 3 predictions and negative-stain EM analyses of NifEN complexed with upstream (NifB) and downstream (NifH) assembly partners, reveal a tunnel linking NifEN with NifB and NifH, with NifEN serving as a dynamic hub that coordinates L-cluster reception, maturation and delivery via conformation-gated metallocluster trafficking.

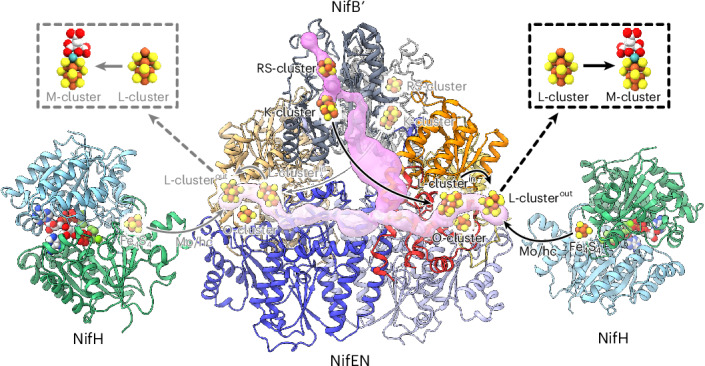

## Abstract (web summary)

Structural insights into the assembly of the complex nitrogenase cofactor are scarce. Now, cryo-EM and AlphaFold analyses of NifEN, which converts the precursor (L-cluster) to a mature cofactor (M-cluster), are reported, uncovering a dynamic tunnel for L-cluster trafficking between assembly partners.

## Main

**Fig. 1:**
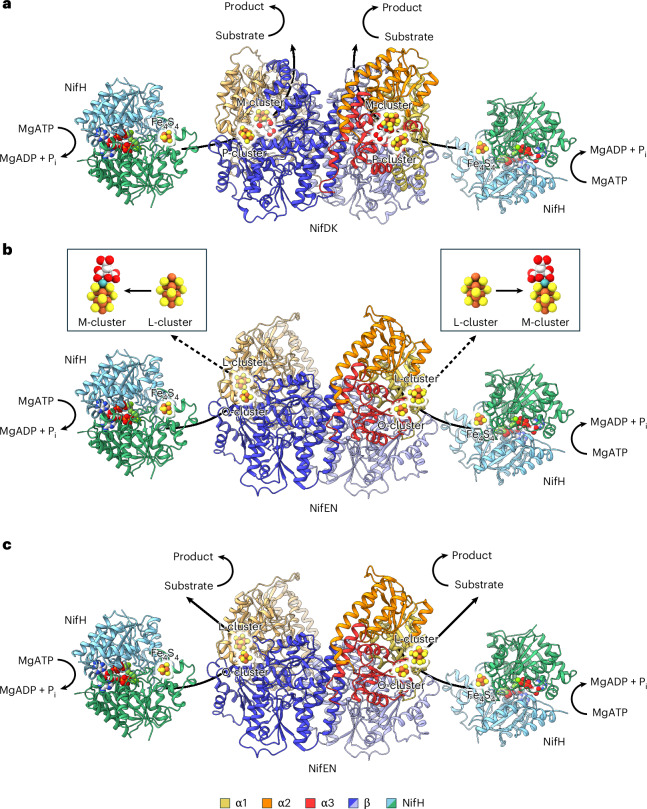
Comparison of NifDK- and NifEN-based catalytic or biosynthetic systems. **a**, The two-component Mo nitrogenase comprises a reductase component (NifH) and a catalytic component (NifDK). This system permits ATP-dependent electron transfer from the [Fe_4_S_4_] cluster (of NifH) through the [Fe_8_S_7_] P-cluster to the [(*R*-homocitrate)MoFe_7_S_9_C] M-cluster (of NifDK) for the reduction of substrates at the active-site cofactor (M-cluster). MgATP, magnesium adenosine triphosphate; MgADP, magnesium adenosine diphosphate; P_i_, inorganic phosphate. **b**, The two-component cofactor biosynthetic system comprises a Mo/homocitrate insertase (NifH) and a cofactor maturase (NifEN). This system facilitates an ATP- and reductant-dependent, NifH-mediated insertion of Mo/homocitrate into the [Fe_8_S_9_C] L-cluster on NifEN, which transforms the all-Fe precursor (L-cluster) into a mature cofactor (M-cluster). **c**, The two-component nitrogenase homologue comprises a reductase component (NifH) and a catalytic component (NifEN). This system permits ATP-dependent electron transfer from the [Fe_4_S_4_] cluster (of NifH) through the [Fe_4_S_4_] O-cluster to the [Fe_8_S_9_C] L-cluster (of NifEN) for the reduction of substrates at the active-site cofactor (L-cluster). In all diagrams, proteins are shown in ribbon presentation, with subunits coloured as indicated in the figure. Clusters and nucleotides are shown as ball-and-stick models, with atoms coloured as follows: Fe, orange; S, yellow; Mo, cyan; C, light grey; Mg, green; O, red; Al, dark grey; F, light blue; P, dark orange. The figure was generated with PDB entries 1N2C^12^ and 3PDI^26^.

Nitrogenasecatalyses the ambient reduction of atmospheric N_2_ to bioavailable NH_3_ in a reaction that parallels the industrial ammonia-producing Haber–Bosch process^1–6^. It can also reduce C_1_ substrates (for example, CO) to hydrocarbons (for example, C_3_H_8_) in a reaction that mirrors the industrial carbon-fuel-producing Fischer–Tropsch process under ambient conditions^7–11^. A two-component enzyme, the classic molybdenum (Mo)-dependent nitrogenase from *Azotobacter vinelandii*, a diazotrophic bacterium, consists of a γ_2_-dimeric reductase component (designated NifH) and an α_2_β_2_-tetrameric catalytic component (designated NifDK) (Fig. 1a)^1–3,12,13^. Catalysis by the Mo-nitrogenase occurs through repeated association and dissociation between its two components, which permits adenosine triphosphate (ATP)-dependent, inter-protein electron transfer from the [Fe_4_S_4_] cluster of NifH, via the P-cluster ([Fe_8_S_7_]), to the M-cluster ([(*R*-homocitrate)MoFe_7_S_9_C]) of NifDK, where substrate reduction takes place (Fig. 1a)^12–15^.

**Fig. 2:**
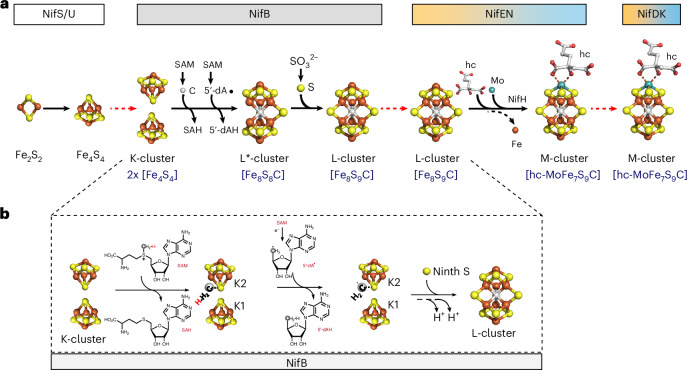
Biosynthesis of the M-cluster. **a**, Biosynthesis of the M-cluster begins with the stepwise formation of [Fe_2_S_2_] and [Fe_4_S_4_] clusters on NifS/U, followed by transfer of an [Fe_4_S_4_] cluster pair (K-cluster) to NifB and its transformation into an [Fe_8_S_9_C] cluster (L-cluster) via radical SAM chemistry. Subsequently, the L-cluster is transferred to NifEN, where it is matured into an [(*R*-homocitrate)MoFe_7_S_9_C] cofactor (M-cluster) upon NifH-mediated insertion of Mo/homocitrate, before delivery of the M-cluster to its final binding site in NifDK. hc, homocitrate. **b**, Proposed pathway of radical SAM-dependent carbide insertion on NifB, which involves methyltransfer from 1 equiv. of SAM to a sulfur atom of the K-cluster in an S_N_2-type reaction. This step is followed by hydrogen atom abstraction from the K-cluster-bound methyl group by a 5’-dA^•^ radical derived from the homolytic cleavage of a second equivalent of SAM, which initiates the radical-dependent coupling and rearrangement of the K-cluster (2 × [Fe_4_S_4_]) into an L-cluster ([Fe_8_S_9_C]) concomitant with the incorporation of an interstitial carbide and a ninth sulfide. Clusters are depicted in ball-and-stick representation, with atoms coloured as in Fig. 1. 5’-dA•, 5’-deoxyadenosyl radical; 5’-dAH, 5’-deoxyadenosine; SAH, *S*-adenosyl-L-homocysteine. The figure was generated using PyMOL^52^ with PDB entries 3U7Q^14^ and 3PDI^26^.

Biosynthesis of the M-cluster (Fig. 2), a high-nuclearity metallocofactor that is biologically important and chemically unprecedented, is arguably one of the most complex processes in metalloprotein biochemistry^16,17^. This process begins with the sequential formation of small [Fe_2_S_2_] and [Fe_4_S_4_] building blocks by NifS (a cysteine desulfurase) and NifU (an FeS-cluster assembly scaffold). Subsequently, an [Fe_4_S_4_] cluster pair (designated K-cluster) is transferred from NifU to NifB (a radical *S*-adenosyl-L-methionine (SAM) enzyme), where it undergoes radical SAM-dependent conversion into an [Fe_8_S_9_C] cofactor core/precursor (designated L-cluster) that is structurally nearly indistinguishable from the M-cluster except for the presence of a terminal Fe atom in place of Mo/homocitrate^18–22^. The L-cluster is then transferred from NifB to NifEN (a cofactor assembly protein) and matured into an M-cluster via NifH-mediated substitution of the terminal Fe atom with Mo/homocitrate^23–26^. This event is followed by transfer of the M-cluster from NifEN to the cofactor binding site within a P-cluster-replete, yet M-cluster-depleted apo-NifDK, resulting in the formation of a P- and M-cluster-replete holo-NifDK^27^.

Sharing substantial homology with NifDK in its primary sequence, tertiary structure and associated metalloclusters^26,28–30^, NifEN plays a pivotal role in M-cluster biosynthesis, receiving the L-cluster from its upstream partner NifB and interacting with its downstream partner NifH to facilitate the L → M-cluster conversion (Fig. 1b). When paired with NifH, NifEN can also mimic NifDK in catalysing N_2_ reduction at the active-site L-cluster (Fig. 1c) under both in vitro and in vivo conditions, further highlighting the structural–functional homology between NifEN and NifDK while pointing to the former as a plausible evolutionary predecessor to the latter^30,31^. The multifunctionality of NifEN makes it an appealing target for structure–function studies; however, detailed structural information remains limited, largely due to the difficulty of capturing the highly dynamic and transient processes NifEN mediates during M-cluster assembly.

In this work, we use single-particle cryogenic electron microscopy (cryo-EM) to elucidate the structural basis of the function of NifEN in nitrogenase cofactor assembly. Integrated with AlphaFold 3 predictions, negative-stain EM analyses and mutational studies, our data reveal conformational changes associated with L-cluster incorporation and identify a tunnel linking NifEN with its assembly partners NifB and NifH. Together, these findings establish NifEN as a dynamic hub that coordinates L-cluster reception, delivery, and maturation through conformation-gated metallocluster trafficking, thereby providing an important framework for future studies of nitrogenase biosynthesis, catalysis and evolution.

## Results

### Expression of *A. vinelandii* NifEN in *Escherichia coli*

Building on our recent success in the heterologous expression of nitrogenase proteins in *E. coli*^31,32^, we co-expressed the *A. vinelandii nifE*,*N* genes with the *A. vinelandii nifB* and *Methanosarcina acetivorans nifS3*,*U3* genes in this non-diazotrophic host, which enabled isolation of a NifEN species (*Ec*NifEN) that was equivalent to its native counterpart (*Av*NifEN) expressed in a *nifHDK*-deletion strain of *A. vinelandii*^33^. Biochemical and spectroscopic analyses demonstrated that *Ec*NifEN was 70% active in a cofactor maturation assay and displayed an L-cluster-specific, *g* = 1.94 EPR (electron paramagnetic resonance)^33^ signal of 66% intensity as compared to *Av*NifEN (Supplementary Fig. 1); both results are consistent with an accumulation of L-clusters on NifEN in the absence of NifH (the L-cluster maturase) and NifDK (the M-cluster receptor). The incomplete L-cluster content of *Ec*NifEN accentuates the specific challenge for metalloprotein assembly, where the biosynthetic flow of metalloclusters is often outpaced by that of the protein scaffolds, particularly given the elevated difficulty of expressing a complex metalloenzyme (such as nitrogenase) with a full complement of metallocentres in a foreign host (such as *E. coli*). At the same time, such a partial occupancy of L-clusters suggests a coexistence of apo (containing only the permanent O-cluster) and holo (containing both the permanent O-cluster and the transient L-cluster) conformations in *Ec*NifEN, which presents an excellent opportunity for us to use cryo-EM to capture dynamic biosynthetic snapshots before (apo) and after (holo) the transfer of the L-cluster from NifB to NifEN.

### Cryo-EM analysis of apo-NifEN and holo-NifEN^in^

**Fig. 3:**
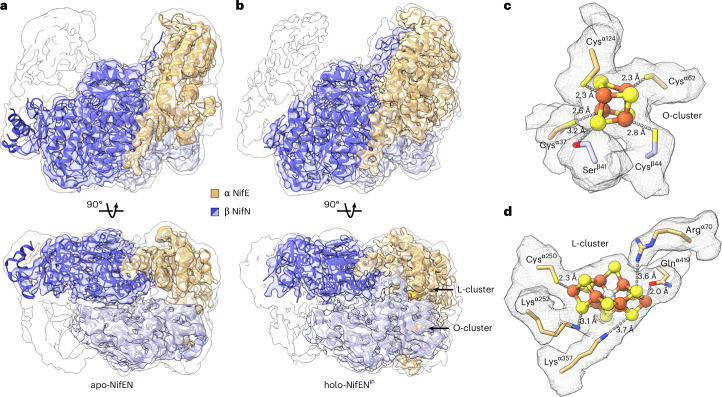
Cryo-EM structures of apo-NifEN and holo-NifEN^in^. **a**,**b**, Structures of apo-NifEN (**a**) and holo-NifEN^in^ (**b**), displayed with the molecular two-fold axis aligned vertically (upper) and rotated 90° (lower). Shown are transparent density maps with their respective cartoon representations as insets and the unmasked map prior to particle subtraction of the unresolved NifE (α) subunit as silhouette. Density maps are displayed at the same threshold for both apo-NifEN and holo-NifEN^in^. Subunits are coloured as indicated in the figure. The L- and O-clusters in holo-NifEN^in^ are shown in ball-and-stick presentation. **c**,**d**, Close-up views of the O-cluster (**c**) and L-cluster (**d**), overlaid with density maps. Both clusters are shown in ball-and-stick representation, with side chains of the ligands and the nearby hydrogen-bonding residues shown in stick presentation. The side chains are coloured according to the subunits in which they reside, and the atoms are coloured as follows: Fe, orange; S, yellow; N, blue; O, red.

Indeed, our cryo-EM analyses revealed the presence of both apo- and holo-conformations (designated apo-NifEN and holo-NifEN^in^, respectively) in the heterologously expressed *Ec*NifEN. Maps of apo-NifEN and holo-NifEN^in^ verified these proteins as α_2_β_2_ tetramers at lower map thresholds, with αβ_2_ trimers resolved to overall resolutions of 3.72 and 3.62 Å, respectively (Fig. 3a,b, Supplementary Fig. 2 and Supplementary Table 1). For both maps, limited local resolution in one α-subunit region prevented reliable model building. Accordingly, this region was masked out during the final local refinement. The angular distribution plots of particles for both maps show that there are hot spots where the particles are well aligned (Supplementary Fig. 3a), although the preferred orientation is modest and does not affect the overall quality of the map (Supplementary Note 1 provides details). In general, the α-subunit of holo-NifEN^in^ appeared to be stabilized via binding of the L-cluster and was consequently resolved to higher resolutions than the α-subunit of apo-NifEN, whereas the α-subunit of apo-NifEN appeared to be dynamic and lacked enough high-resolution density to sufficiently model sections of the subunit (Supplementary Figs. 3b, 4 and 5).

The α (NifE) and β (NifN) subunits of both NifEN species are composed of three domains: α1, α2, α3 and β1, β2, β3 (Supplementary Figs. 4 and 6a,b). In the case of apo-NifEN, the quality of the map prevented modelling of the O-cluster and its ligands, although some map signal was observed at the O-cluster site between the NifE and NifN subunits (Supplementary Fig. 3c). Given the expected high occupancy of the permanent O-cluster (Fig. 1b), the low map density probably originates from the presence of this cluster in a highly dynamic region (Supplementary Fig. 5 and Supplementary Video 1). In the case of holo-NifEN^in^, however, the quality of the map permitted modelling of the O-cluster, with its four Fe atoms ligated by three Cys residues from the NifE subunit (Cys^α37^, Cys^α62^, Cys^α124^) and additionally interacting with two residues from the NifN subunit (Ser^β41^, Cys^β44^) via hydrogen bonds (Fig. 3c). Similarly, the strong density of the L-cluster allowed for identification of the analogous, yet distinct location and coordination of this cluster in holo-NifEN^in^ (Fig. 3d) as compared to those of its M-cluster counterpart in holo-NifDK (Supplementary Fig. 7). Specifically, one terminal Fe atom of the L-cluster is ligated by Cys^α250^ of the NifE subunit, corresponding to Cys^α275^ of the NifD subunit that ligates the terminal Fe atom of the M-cluster, whereas the opposite terminal Fe atom of the L-cluster is ligated by Gln^α419^ of the NifE subunit, a much weaker ligand than His^α442^ of the NifD subunit that ligates the terminal Mo atom of the M-cluster (Fig. 3d and Supplementary Fig. 7)^14^. Additionally, the Gln^α419^-ligated Fe-end of the L-cluster does not have an equivalent to Trp^α444^ of the NifD subunit, which locks the M-cluster in place by its bulky, aromatic side chain upon a swap in position with His^α442^ (Fig. 3d and Supplementary Fig. 7)^27^. Instead, the L-cluster is surrounded by three residues in moderate/long hydrogen-bonding distances to three of its sulfur atoms (Fig. 3d and Supplementary Fig. 7), with each residue contributed by one of the three domains of NifE (Arg^α70^ (α1-domain), Lys^α252^ (α2-domain) and Lys^α357^ (α3-domain)).

**Fig. 4:**
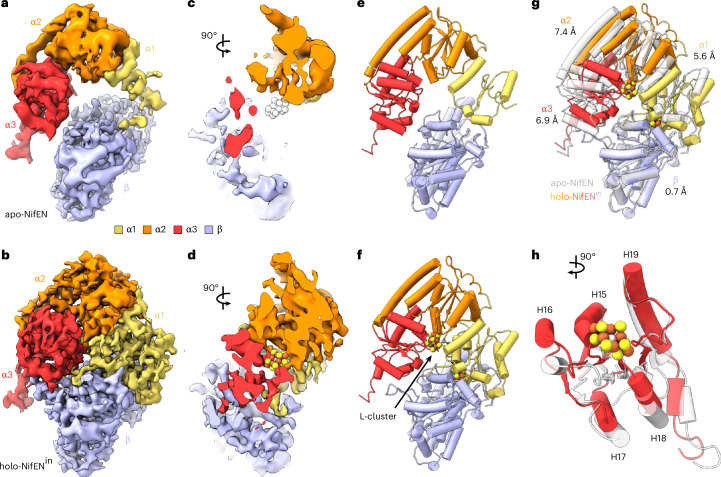
Comparison of the αβ-pairs of apo-NifEN and holo-NifEN^in^. **a**–**f**, Anterior views of EM maps (**a**,**b**), vertical slices through the side of the density maps (**c**,**d**) and cartoon representations (**e**,**f**) of apo-NifEN (**a**,**c**,**e**) and holo-NifEN^in^ (**b**,**d**,**f**). The colouring of the three domains of the α- and β-subunits is indicated in the figure. **g**,**h**, Superimposed αβ-pairs (**g**) and α3-domains (**h**) of apo-NifEN and holo-NifEN^in^, with apo-NifEN rendered in transparent grey. The L- and O-clusters are depicted as ball-and-stick models, with the same colouring as that in Fig. 3. Note that the L-cluster, rendered in transparent grey, is placed in apo-NifEN upon alignment with holo-NifEN^in^ (**c**). The average Cα deviations of the three domains of the NifE (α)- and NifN (β)-subunits are indicated in **g**. The key helices (H15–H19) of the α3-domain are highlighted in **h**.

Strikingly, a comparison of the maps of apo-NifEN and holo-NifEN^in^ revealed the presence of a much wider tunnel in the former than in the latter (Fig. 4a,b). Spanning across the entire NifEN dimer, the tunnel is walled off by both NifE and NifN subunits (Fig. 4c,d). The Cα deviations derived from a superimposition of apo-NifEN and holo-NifEN^in^ (Fig. 4e–g, Supplementary Fig. 8a and Supplementary Video 2) illustrate a minor movement of the three domains of the NifN subunit (by 0.6–0.7 Å) that contrasts with the major movement of all three domains of the NifE subunit (by 5.6–7.4 Å) towards the tunnel, resulting in an overall contraction of the tunnel in holo-NifEN^in^. Of particular note, there is a substantial movement of several helices in the α3-domain of holo-NifEN^in^ (H15, H16, H17, H18, H19) by up to ~10 Å towards the L-cluster binding site (Fig. 4h), thereby partially closing the tunnel and nestling the L-cluster in a network of hydrogen bonds with one terminal Fe ligated by a strong Cys^α250^ ligand and the other coordinated by a weak Gln^α419^ ligand (Fig. 3d). Overall, the structure of NifEN becomes substantially more ordered upon incorporation of the L-cluster. Yet, the notable absence of a second strong ligand (for example, an equivalent to the His^α442^ of NifD) and other spatial restrictions (for example, an equivalent to the Trp^α444^ of NifD) at one Fe-end of the L-cluster renders its coordination in holo-NifEN^in^ much more flexible than that of the M-cluster in holo-NifDK, pointing to a distinct possibility for the L-cluster to translocate to another site for its maturation into an M-cluster.

### Paired comparison of NifEN (in/out) and NifDK (apo/holo)

**Fig. 5:**
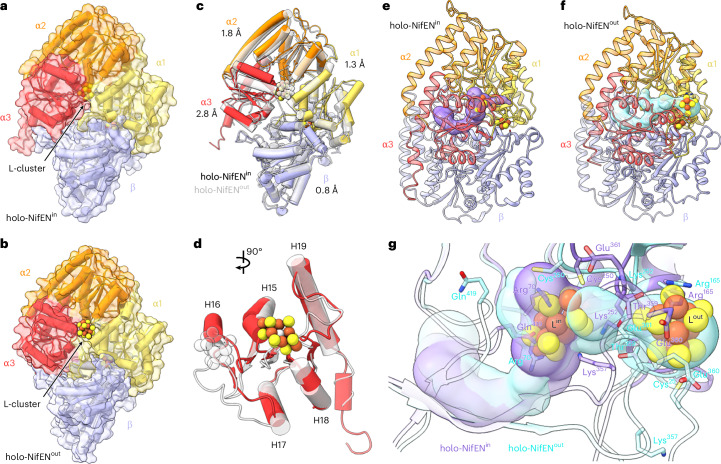
Comparison of the αβ-pairs of holo-NifEN^in^ and holo-NifEN^out^. **a**,**b**, Transparent surface with cartoon representations of holo-NifEN^in^ (**a**) and holo-NifEN^out^ (**b**) as insets. The colouring of the three domains of the α- and β-subunits follows the scheme used in Fig. 4. **c**, Superimposition of the αβ-pairs of holo-NifEN^in^ and holo-NifEN^out^, with holo-NifEN^out^ rendered in transparent grey. The average Cα deviations of the three domains of the α- and β-subunits are indicated. **d**, Superimposition of the α3-domains of holo-NifEN^in^ and holo-NifEN^out^, with holo-NifEN^out^ rendered in transparent grey. The key helices (H15–H19) of the α3-domain are indicated. **e**,**f**, Identification of potential tunnels for L-cluster trafficking in holo-NifEN^in^ (**e**) and holo-NifEN^out^ (**f**). Tunnels are depicted in purple (holo-NifEN^in^) and cyan (holo-NifEN^out^), with the αβ-pairs of the two proteins colour-coded as in **a**–**c**. **g**, Close-up view of the overlaid tunnels in holo-NifEN^in^ and holo-NifEN^out^. The key residues interacting with the L-cluster in holo-NifEN^in^ and holo-NifEN^out^ are coloured purple and cyan, respectively. The L- and O-clusters are shown in ball-and-stick presentation, with the same colouring as in Fig. 3. Tunnel analysis was performed using MOLEonline^35,36^, and the figure was generated with PDB entries 3PDI^26^ for holo-NifEN^out^ and 9ONK for holo-NifEN^in^.

Consistent with this suggestion, our previous X-ray crystallographic analysis revealed another holo-NifEN conformation (designated holo-NifEN^out^)^26^, with the L-cluster coordinated by a Cys^α25^ ligand at a surface-exposed location (Fig. 5a,b and Supplementary Fig. 9a). A structural superimposition of holo-NifEN^out^ and holo-NifEN^in^ (Fig. 5c, Supplementary Fig. 8b and Supplementary Video 3) shows an overall root-mean-square deviation (RMSD) of 0.9 Å, suggesting a high degree of similarity between the two holo-NifEN conformations, namely in/out. However, there is a clear structural rearrangement in the α3-domain, as indicated by an average Cα deviation of 2.8 Å when the corresponding domains in holo-NifEN^out^ and holo-NifEN^in^ are compared with each other (Fig. 5c and Supplementary Fig. 8b). Most notably, one helix (H16) in the α3-domain, which shifts by 10.1 Å towards the interior L-cluster binding site in holo-NifEN^in^ relative to its position in apo-NifEN (Fig. 4h and Supplementary Fig. 8a), swings 6.7 Å away from the interior L-cluster site in holo-NifEN^out^ relative to its position in holo-NifEN^in^ (Fig. 5d and Supplementary Fig. 8b), thereby partially opening the path leading from the interior L-cluster binding pocket (in holo-NifEN^in^) to the surface location of this cluster (in holo-NifEN^out^) (Fig. 5e–g, Supplementary Fig. 10a,b and Supplementary Video 4).

Interestingly, a structural overlay of apo-NifDK^27^ and holo-NifDK^14^ unveils a path that is strikingly similar to that identified through a structural superimposition of the structures of holo-NifEN^out^ and holo-NifEN^in^ (Supplementary Fig. 8c). Showing an overall RMSD of 0.4 Å, apo-NifDK and holo-NifDK are structurally highly similar; however, as observed in the structural comparison of holo-NifEN^in^ and holo-NifEN^out^, there is a notable, average Cα deviation of the α3-domain by 3 Å when apo-NifDK and holo-NifDK are compared with each other (Supplementary Fig. 8c). Moreover, consistent with the substantial motion of one α3-domain helix (H16) in holo-NifEN^out^ relative to that in holo-NifEN^in^, the corresponding α3-domain helix (H16) in apo-NifDK moves 7.8 Å away from the interior M-cluster binding site identified in holo-NifDK (Supplementary Fig. 8c and Supplementary Video 5) and, consequently, opens a path leading to this binding pocket. Given the necessity of NifEN to host multiple biosynthetic events, the path identified in NifEN is possibly bidirectional, allowing the L-cluster to migrate from the interior binding site to the surface location (that is, from in to out; Supplementary Fig. 9c) for its transformation into an M-cluster upon receipt of Mo/homocitrate from NifH and, in the opposite direction, allowing the M-cluster to migrate from the protein surface back to its interior binding site (that is, from out to in; Supplementary Fig. 9c) prior to transfer of the M-cluster from NifEN to its final location in NifDK. In contrast, the corresponding path identified in NifDK is probably unidirectional, allowing the M-cluster to migrate solely from the protein surface to its targeted binding site (that is, from out to in; Supplementary Fig. 9d). The lower affinity of the cofactor binding site in NifEN would enable the facile in-and-out of the cofactor during biosynthesis, with one strong terminal ligand (in: Cys^α250^; out: Cys^α25^) assisting the relay of the cofactor between different binding sites, whereas the higher affinity of the cofactor binding site in NifDK would serve to secure the cofactor at its binding site, with a pair of terminal ligands (Cys^α272^, His^α442^) and other key residues (Trp^α444^) locking the cofactor in place to carry out its catalytic function.

### AlphaFold 3-predicted NifEN–NifB and NifEN–NifH interactions

The paired structural comparison of apo-NifEN/holo-NifEN^in^ and holo-NifEN^in^/holo-NifEN^out^ points to a plausible sequence of events that begins with a global conformational change of apo-NifEN (involving all three α-domains) upon incorporation of the L-cluster, and continues with a more localized conformational change (involving only the α3-domain) that accompanies the migration of the L-cluster between its interior binding site (in holo-NifEN^in^) and the surface location (in holo-NifEN^out^). Such a scenario begs the question of where NifB, the upstream biosynthetic partner of NifEN, deposits the L-cluster to allow its initial entry into the open tunnel in apo-NifEN, given the presence of a front entrance (near the surface location of the L-cluster in holo-NifEN^out^) and a back entrance (at the opposite end of the L-cluster in holo-NifEN^out^) of this through-tunnel. To probe the site of interactions between NifEN and NifB, we turned our attention to a NifEN-B′ fusion protein, in which the C terminus of NifN is fused with the N terminus of NifB′, a truncated NifB generated upon removal of the non-essential NifX domain (Supplementary Figs. 11 and 12). Biochemical and spectroscopic analyses demonstrated the ability of NifEN-B′ to perform the same function in cofactor assembly as that accomplished by the individual NifEN and NifB proteins (Supplementary Fig. 1); moreover, they led to the assignment of L- and K-clusters on the NifEN and NifB′ entities, respectively, with the K-clusters capable of undergoing conversion to additional L-clusters upon incubation of NifEN-B′ with SAM (Supplementary Fig. 1). Together, these results establish the feasibility for us to use the fusion strategy to reduce the uncertainty in predicting the site of interaction between NifEN and NifB.

**Fig. 6:**
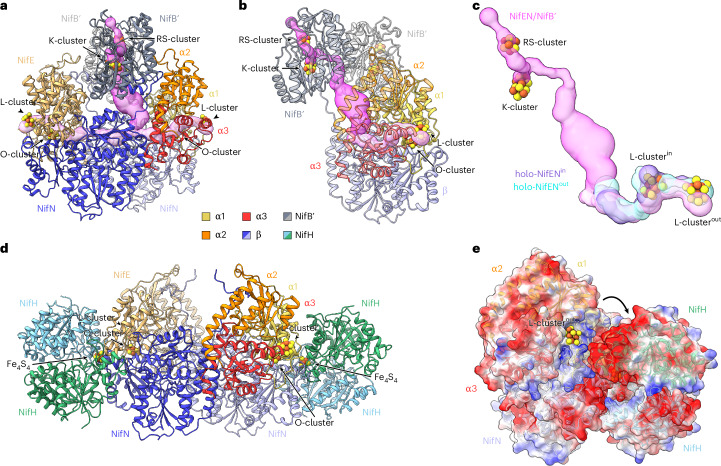
AlphaFold 3 predictions of the NifEN-B′ fusion and the NifEN/NifH complex. **a**,**b**, Structural prediction of the complete (**a**) and half (**b**) NifEN-B′ fusion, shown in cartoon representation with domains and subunits colour-coded as indicated in the figure. Tunnel analysis was performed using MOLEonline^35,36^ and reveals a continuous tunnel spanning NifB′ and NifEN, encompassing the RS-, K- and L-clusters (pink). The locations of the metal clusters were predicted upon superimposition of the NifEN-B′ fusion model with known structures of NifEN (PDB 3PDI)^26^ and NifB (PDB 7JMB)^22^ (Supplementary Fig. 13). **c**, Superimposition of the tunnels identified in NifEN-B′ (pink), holo-NifEN^in^ (purple) and holo-NifEN^out^ (cyan). Metal clusters along the tunnel paths are shown as ball-and-stick models. **d**, Structural prediction of the NifEN/NifH complex, shown in cartoon representation with subunits colour-coded as indicated in the figure. The locations of the metal clusters were predicted upon superimposition of the NifEN/NifH complex model with known structures of NifEN (PDB 3PDI)^26^ and NifH (PDB 1N2C)^12^ (Supplementary Fig. 14). **e**, Electrostatic surface representation of the half NifEN/NifH complex, with negatively and positively charged regions shown in red and blue, respectively. The transparent surface is overlaid with a cartoon representation of the complex, with the surface-exposed L-cluster (L-cluster^out^) shown as a ball-and-stick model.

Building on this premise, we then used AlphaFold 3^34^ to perform a structural prediction of NifEN-B′, which yielded a high-confidence model with overall pTM and ipTM scores of 0.76 and 0.8, respectively (Supplementary Fig. 13a). The good quality of the model is further supported by RMSD values of <1 Å upon superimposition of the NifEN and NifB′ entities of the NifEN-B′ model with the available X-ray crystal structures of NifEN (from *A. vinelandii*)^26^ and NifB (from *M. thermoautotrophicum*)^22^, respectively (Supplementary Fig. 13b,c). Interestingly, the AlphaFold 3 model revealed a symmetric binding of two NifB′ entities—each fused to a NifN subunit—within the valley-shaped tetrameric NifEN complex, wherein two NifN subunits form the base and two NifE subunits create the slopes of the valley (Fig. 6a and Supplementary Fig. 13a). The notable degree of similarity between the AlphaFold 3 models and the crystal structures facilitated a high-confidence placement of the metal clusters in the NifEN and NifB entities of NifEN-B′ (Fig. 6a and Supplementary Fig. 13a), thereby permitting further exploration of potential funnels linking these cluster intermediates during the cofactor assembly process. Interestingly, a continuous tunnel spanning NifB′ and NifEN entities and encompassing the RS-, K- and L-clusters (Fig. 6a,b) was identified using the software MOLEonline^35,36^. Perhaps even more strikingly, the NifEN segment of this tunnel overlaps well with the tunnels identified in holo-NifEN^in^ and holo-NifEN^out^ (Fig. 6c; also Fig. 5g), pointing to a possible metallocluster trafficking pathway for the L-cluster to exit NifB upon SAM-dependent K → L-cluster conversion (Fig. 2), enter NifEN, and route via the interior binding site (in holo-NifEN^in^) to the surface location (in holo-NifEN^out^), which enables an easier access of the NifEN-bound L-cluster by NifH for the subsequent L → M cluster maturation upon Mo/homocitrate insertion.

Consistent with this argument, the structural prediction of the NifEN/NifH complex by AlphaFold 3, coupled with the structure-based assignment of metalloclusters in the model, positioned NifH near the surface-exposed L-cluster in NifEN (Fig. 6d,e and Supplementary Fig. 14a,b). The overall pTM and ipTM scores of 0.87 and 0.85, respectively, of this model (Supplementary Fig. 14b) align well with the RMSD values of ~1 Å upon superimposition of the NifEN and NifH components of the model with the available X-ray crystallographic structures of NifEN^26^ and NifH^12^, respectively, from *A. vinelandii* (Supplementary Fig. 14b), collectively pointing to the accuracy of the prediction. Moreover, the high degree of similarity of the superimposed models/structures of NifEN/NifH permitted a high-confidence placement of the metal clusters within the NifEN/NifH complex (Fig. 6d and Supplementary Fig. 14a), resulting in a structural model with a strong resemblance to the crystal structure of the homologous, AlF_4_^−^-stabilized NifDK–NifH complex (Supplementary Fig. 14c)^12^ with respect to the arrangement of their corresponding components and associated metalloclusters.

Notably, the structural predictions suggest that NifH interacts with NifEN at a face that does not overlap with the face (or valley) interacting with NifB, thereby allowing NifEN to interact with NifB and NifH, its upstream and downstream partners, respectively, in cofactor biosynthesis without mutual interference. The electrostatic surface representation of the NifEN/NifH complex illustrates that the L-cluster is situated in a positively charged region of NifE immediately next to the opening of the tunnel (Fig. 6e). The NifH docked in close proximity, on the other hand, contains a negatively charged patch at the surface (Fig. 6e) and is thus well-poised to interact with the positively charged NifE patch housing the L-cluster to facilitate its subsequent maturation into an M-cluster. The conformational rearrangement needed for such an interaction, possibly triggered by loading of NifH with Mo/homocitrate, enables the insertion of Mo/homocitrate by NifH into the NifEN-bound L-cluster. This event is probably followed by a conformational change that results in the dissociation of NifH from NifEN concomitant with a translocation of the M-cluster from the surface of NifEN, via the positively charged path (the front path), to its binding site within NifEN, a scenario indicated by the placement of the M-cluster upon a structural superimposition of holo-NifEN^out^ and holo-NifDK (Supplementary Fig. 10c). Conceivably, although the M- and L-clusters on NifEN could share the same ligand (Cys^α250^) at one Fe-end of the cluster, the presence of Mo/homocitrate at the opposite end of the M-cluster necessitates different ligands (Supplementary Fig. 7) for its coordination within NifEN prior to its release to NifDK.

### Evidence for proposed L-cluster trafficking during assembly

**Fig. 7:**
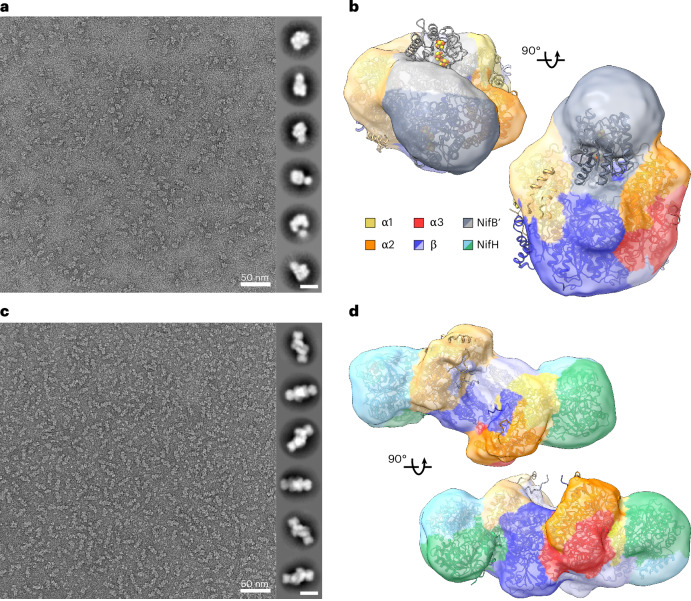
Negative stain of the NifEN-B′ fusion and the NifEN/NifH complex. **a**, A representative negative-stain micrograph of a dataset of 167 images (scale bar, 50 nm) and 2D classes of the NifEN**-**B′ fusion (insets: scale bars, 10 nm). **b**, The AlphaFold 3 model of the NifEN-B′ fusion was rigid body fit into the refined negative-stain 3D reconstruction. **c**, A representative negative-stain micrograph of a dataset of 52 images (scale bar, 50 nm) and 2D classes of the MgADP•AlF_4_^−^-stabilized NifEN/NifH complex (insets: scale bars, 10 nm). **d**, The AlphaFold 3 model of the NifEN/NifH complex was rigid body fit into the refined negative-stain 3D reconstruction.

To obtain experimental support for our proposed NifEN–NifB and NifEN–NifH interactions, we performed negative-stain EM analyses of the NifEN–B’ fusion protein and the MgADP•AlF_4_^−^-stabilized NifEN/NifH complex. The two-dimensional (2D) classification and three-dimensional (3D) refinement of the NifEN–B′ fusion from negative-stain EM reveal that the fused NifB′ domain occupies a position above the central valley formed by the NifEN scaffold (Fig. 7a,b and Supplementary Fig. 15a). This arrangement is in close agreement with the AlphaFold 3 model (Fig. 6a), although the 3D negative-stain reconstruction of NifB′ in the valley appears somewhat off-centre, which probably reflects a dynamic and transient NifB–NifEN interaction that prevents NifB from remaining in contact with NifEN for an extended period. Similarly, negative-stain EM analysis of the MgADP•AlF_4_^−^-stabilized NifEN/NifH complex (Fig. 7c,d and Supplementary Fig. 15b) strongly corroborates the AlphaFold 3 prediction (Fig. 6d), showing that NifH engages NifEN at a distinct face separate from the valley that accommodates NifB. Only in this case, the 3D negative-stain reconstruction and AlphaFold 3 model of the stabilized NifEN/NifH complex align almost perfectly, consistent with the tendency of AlphaFold to predict the most stable, low-energy conformation. Together, these observations highlight the structural complementarity among NifB, NifEN and NifH, and underscore the predictive power of the AlphaFold 3 models in capturing the organization of these nitrogenase assembly intermediates.

We then examined the proposed route of L-cluster trafficking through site-directed mutagenesis of NifEN, targeting the predicted ligands at the NifB–NifEN interface (designated NifEN^IF^), along the back entryway (designated NifEN^BE^), at the back opening of the funnel (designated NifEN^BO^), around the interior L-cluster binding site (designated NifEN^Int^), and at the front opening of the funnel (designated NifEN^FO^), facing the NifEN–NifH interface (see Supplementary Figs. 16–19 for details). The NifEN^BE^, NifEN^BO^ and NifEN^Int^ variants carry Arg-to-Ala substitutions that disrupt electrostatic and hydrogen-bonding interactions with the L-cluster, thereby impairing cluster transport, whereas the NifEN^FO^ variant carries Cys-to-Ala substitutions that eliminate the thiol ligands essential for L-cluster coordination and maturation.

Biochemical and spectroscopic analyses demonstrated a complete loss of L-cluster content in the NifEN^IF^ (Supplementary Fig. 17), NifEN^BE^ and NifEN^BO^ (Supplementary Fig. 19) variants, reflecting a serious obstruction of L-cluster delivery upon disruption of the contact between NifB and NifEN or removal of the transient ligands guiding the L-cluster all the way to the back entrance of the tunnel. In contrast, the NifEN^Int^ and NifEN^FO^ variants (Supplementary Fig. 19) were still able to retain the L-cluster, although their L-cluster content was clearly reduced compared with that of their wildtype counterpart, highlighting a distinct role of these residues in facilitating L-cluster delivery across NifEN upon receipt from NifB. Notably, there is a pronounced polar effect of mutations on the L-cluster content of NifEN, with the strongest influence observed near the NifB–NifEN interface and the weakest near the NifEN–NifH interface. This pattern provides strong support for our proposed directionality of the L-cluster delivery channel, running from the NifB–NifEN interface via the interior L-cluster binding site towards the NifEN–NifH interface. Moreover, the strong disruption of L-cluster maturation caused by mutation of the exterior cluster binding site at the NifEN–NifH interface suggests that the L-cluster transitions from the interior to the exterior site for conversion to the M-cluster upon NifH interaction. Conversely, our previous finding that an engineered NifEN variant containing a high-affinity M-cluster site can retain the matured cofactor^37^ supports the possibility of a reverse movement of the M-cluster back to a protected site in NifEN, prior to its delivery to NifDK.

## Conclusions

**Fig. 8:**
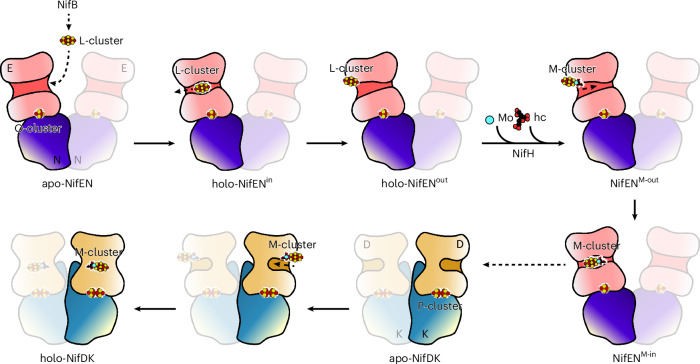
Schematic presentation of the proposed biosynthetic events on NifEN and NifDK. The L-cluster is transferred from NifB to the entrance of the L-cluster insertion funnel located in the valley of apo-NifEN, formed with two NifE (α) subunits as the slopes and two NifN (β) subunits as the base. Subsequent trafficking of the L-cluster to the interior binding site (in holo-NifEN^in^), followed by its relocation to the surface-exposed binding site (in holo-NifEN^out^) via a cluster delivery funnel, positions the L-cluster at the face of NifEN that is opposite to the face that interacts with NifB. The relocation of the L-cluster to the surface allows NifH to directly access and insert Mo/homocitrate into the L-cluster, resulting in a surface-bound, mature M-cluster (in NifEN^M-out^) that is subsequently transferred to its interior binding site (in NifEN^M-in^). Upon docking with apo-NifDK, NifEN^M-in^ releases the M-cluster from its interior binding site and thereby allows the M-cluster to relocate back to the surface of NifEN, followed by transfer of the M-cluster from NifEN to the surface of apo-NifDK, and insertion of the M-cluster via the cofactor insertion funnel into its final binding site, culminating in the formation of a cluster-replete holo-NifDK. The proposed cluster delivery tunnel and biosynthetic sequence in NifEN parallel those in NifDK, highlighting the strong similarities between the two proteins in tertiary structure and cluster topology.

Although the AlphaFold 3-based modelling is limited by a lack of consideration of structural rearrangements induced by metallocluster incorporation, they could be effectively paired with structural analyses (as illustrated herein) to probe the dynamic metallocofactor assembly process that is otherwise difficult to access. Together, our experimental and theoretical analyses lead to the proposal of an alternative model wherein a unique L-cluster trafficking pathway connects the biosynthetic events occurring upstream and downstream of NifEN (Fig. 8). Contrary to the previously proposed model wherein NifB and NifH sequentially interact with the same face of NifEN for the initial deposit (by NifB) and the subsequent maturation (by NifH) of the L-cluster on NifEN (Supplementary Fig. 20)^26^, this alternative model depicts a well-coordinated interaction of NifEN with NifB and NifEN at two distinct faces, which could contribute to an improved efficiency of NifEN in hosting the consecutive events in L-cluster maturation and delivery. The global conformational rearrangement involving all three α-domains of NifEN enables an intra-protein transit of the L-cluster en route to the protein surface to receive Mo/homocitrate from NifH, whereas the subsequent, more localized movement of the α3-domain of NifEN accommodates a translocation of the matured M-cluster back to its binding site, prior to its delivery to NifDK, further highlighting the effectiveness of a conformation-gated mechanism that facilitates dynamic metallocluster trafficking during cofactor biosynthesis.

The conformational flexibility of NifEN also has intriguing implications for the evolution of nitrogenase. Ancestral sequence reconstruction studies have suggested that the last common ancestor of the NifDK protein family, which comprise nitrogenase and nitrogenase-like enzymes, probably featured a complex L-cluster-like metal centre at its active site that was coordinated differently from the M-cluster in modern nitrogenases or the L-cluster in methylthio-alkane reductases^38^. In both extant enzymes, the cluster is firmly anchored within an internal binding pocket by one cysteine ligand and one histidine ligand^14,38^, which facilitate their distinct catalytic functions. However, the His ligands are located on different loop regions in the NifDK of nitrogenases and methylthio-alkane reductases (Mar)^38^, suggesting that these strong His ligands were probably absent from the ancestral enzyme and that they later evolved independently to stabilize cluster binding in different enzymes. Notably, ancestral sequence predictions indicate the presence of a Gln residue at the position corresponding to the histidine ligand that locks the cluster in NifDK^14,38^, consistent with the coordination of one end of the L-cluster by a Gln^α419^ residue at the internal binding site of NifEN^in^ (Fig. 3d and Supplementary Fig. 7a). Such a weakened coordination would give the L-cluster sufficient freedom to move to the surface of NifEN^out^ (Fig. 5g and Supplementary Fig. 9a) for its maturation into an M-cluster during biosynthesis. Moreover, it could account for the much lower efficiency of NifEN in catalysing N_2_ reduction than its modern counterpart, an observation that led to the proposal of NifEN as a prototype ancient nitrogenase that gave rise to both the catalytic component and the specialized biosynthetic protein of the extant nitrogenase system^30^. Taken together, the structural insights acquired through this work provide a crucial platform for further investigations into the biosynthesis, catalysis and evolution of the enigmatic nitrogenase enzyme.

## Methods

### General materials and practices

Unless specified otherwise, all chemicals were purchased from Sigma-Aldrich and Thermo Fisher Scientific. All experiments were conducted in a glove box or on a Schlenk line under an Ar atmosphere, with an O_2_ concentration of <3 ppm. All DNA was synthesized and cloned by GenScript and Azenta Life Sciences.

### Cell growth and protein purification

The *E. coli* strain expressing a His-tagged, L-cluster-bound form of *A. vinelandii* NifEN (designated *Ec*NifEN) was constructed as described previously^31^ except for the omission of the genes responsible for the synthesis and maturation of NifH from *A. vinelandii* (that is, *Av nifH*,*M*). The *E. coli* strains expressing His-tagged *Ec*NifEN^IF1^, *Ec*NifEN^IF2^, *Ec*NifEN^BE1^, *Ec*NifEN^BE2^, *Ec*NifEN^BO^, *Ec*NifEN^Int1^, *Ec*NifEN^Int1/2^, *Ec*NifEN^Int2^ and *Ec*NifEN^FO^ variants were constructed the same way as described for the strain expressing *Ec*NifEN, expect that they contained the mutations detailed in Supplementary Figs. 17 and 19 in the genes encoding NifE and/or NifN. Similarly, the *E. coli* strain expressing His-tagged, L-cluster-deficient *Ec*NifEN^apo^ was constructed in the same way as described for the strain expressing *Ec*NifEN, except for omission of the encoding gene for NifB, an essential enzyme for L-cluster synthesis.

Each of the *E. coli* expression strains was grown in 10-litre batches in Luria-Bertani (LB) medium (Difco) supplemented with 50 mM MOPS/NaOH (pH 7.4), 25 mM glucose, 2 mM ferric ammonium citrate, 27 mg l^−1^ kanamycin and 27 mg l^−1^ streptomycin in a BIOFLO 415 fermenter (New Brunswick Scientific) at 37 °C with agitation at 200 r.p.m. and air flow of 10 l min^−1^. When the optical density at 600 nm (OD_6__00_) reached 0.4, the air flow was terminated, the fermenter was purged with N_2_ (ultrahigh purity) at a rate of 1.5 l min^−1^, and the temperature was lowered to 30 °C. Once the temperature reached 30 °C, 27 mM sodium fumarate and 2 mM cysteine were added and the expression of nitrogenase was induced by the addition of 420 µM isopropyl β-D-1-thiogalactopyranoside (IPTG). Protein expression was allowed to continue for 16 h, followed by collection of the cells by centrifugation using a Thermo Fisher Scientific Legend XTR centrifuge. The heterologously expressed, His-tagged *Ec*NifEN or its variant was then purified by immobilized metal affinity chromatography (IMAC) using a method adapted from a previously established protocol^39^, followed by size exclusion chromatography (SEC) using the Cytiva AKTA go system equipped with a Cytiva Superdex 200 Increase 10/300 GL column. A buffer containing 50 mM Tris‒HCl (pH 8.0), 500 mM NaCl and 1% glycerol was used to equilibrate the SEC column and elute *Ec*NifEN or its variant.

Construction of the *A. vinelandii* strain expressing a His-tagged, NifEN-B′ fusion protein (designated *Av*NifEN-B′), in which the NifX domain of NifB (amino acids 156–503) was removed, has been reported previously^40^. This strain, along with *A. vinelandii* strains DJ1162, DJ1143 and DJ1041 expressing His-tagged *Av*NifH, *Av*NifDK^apo^ and *Av*NifEN^33,41^, respectively, was grown in 180-litre batches in Burke’s minimal medium supplemented with 2 mM ammonium acetate in a 200-litre fermenter (New Brunswick Scientific) at 30 °C with agitation at 100 r.p.m. and an air flow of 30 l min^−1^. Cell growth was monitored at OD_436_ using a Spectronic 20 Genesys spectrometer (Spectronic Instruments) and, upon depletion of ammonia, cells were de-repressed for 3 h before being harvested by a flow-through centrifugal harvester (Cepa). Published methods were used to purify His-tagged *Av*NifH, *Av*NifDK^apo^ and *Av*NifEN^33,41^.

### Metal analysis

The metal contents of *Ec*NifEN or its variants, *Av*NifEN and *Av*NifEN-B′, were determined by inductively coupled plasma optical emission spectroscopy (ICP-OES) using an iCAP7000 ICP-OES spectrometer (Thermo Scientific Scientific). Calibration of the equipment was performed by using standard solutions made via dilution of a stock solution of elemental Fe (1 mg ml^−1^). The protein sample was mixed with 100 µl of concentrated sulfuric acid (H_2_SO_4_) and 100 µl of concentrated nitric acid (HNO_3_), followed by heating of this mixture for 30 min at 250 °C. This procedure was repeated until the solution became colourless. Subsequently, the solution was cooled to room temperature and diluted to a total volume of 7.5 ml with 2% HNO_3_, before metal analysis.

### Maturation assays

Each maturation assay contained, in a total volume of 0.9 ml, 25 mM Tris‒HCl (pH 8.0), 0.3 mg of the P-cluster containing, yet M-cluster-deficient *Av*NifDK^apo^, 1.0 mg of NifH, 1.4 mg of *Ec*NifEN, *Av*NifEN, or *Av*NifEN-B′ (with or without 10 µM SAM), 0.5 mM homocitrate, 0.25 mM Na_2_MoO_4_, 2.4 mM ATP, 4.8 mM MgCl_2_, 30 mM creatine phosphate, 24 units of creatine phosphokinase and 20 mM Na_2_S_2_O_4_. Additionally, each assay contained 1.0 atm Ar in the headspace. The reaction was incubated at 30 °C for 60 min and subsequently split into duplicates in two 9.5-ml vials, each containing 1.4 mg of *Av*NifH, 25 mM Tris‒HCl (pH 8.0), 2.5 mM ATP, 5.0 mM MgCl_2_, 30 mM creatine phosphate, 0.125 mg of creatine phosphokinase and 20 mM Na_2_S_2_O_4_ in a total volume of 0.6 ml. Additionally, it contained 0.1 atm C_2_H_2_ and 0.9 atm Ar in the headspace. The reaction mixture was then incubated at 30 °C for 10 min and analysed for product formation as described previously^40^.

### SAM cleavage assays

Each SAM cleavage reaction contained, in a total volume of 0.5 ml, 25 mM Tris–HCl (pH 8), 5% glycerol (vol/vol), 1.7 mg (21 μM) of *Av*NifEN-B′ and 64 µM SAM. The reaction mixture was incubated at 25 °C for 60 min with three intermittent mixings. It was then terminated by filtration through Amicon Ultra 30,000 MWCO centrifugal filters. Trifluoroacetic acid was then added to the reaction mixture at a concentration of 0.14%, followed by analysis of the resultant sample by a Thermo Scientific Dionex Ultimate 3000 UHPLC system equipped with an Acclaim 120 C18 column (4.6 × 100 mm, 5-µm particle size). The column was equilibrated with 98% buffer A (50 mM KH_2_PO_4_, pH 6.6) and 2% buffer B (100% methanol) before each sample injection for at least 5 min. Following sample injection (100 µl per sample), a linear gradient of 2–60% buffer B was applied to the column for 20 min, followed by an isocratic flow with 60% buffer B for 8 min, and a linear gradient of 60–2% buffer B for 4 min. Throughout the run, the flow rate of the buffer was kept at 0.5 ml min^−1^, and the column was kept at 30 °C. Elution of products was monitored at an ultraviolet wavelength of 254 nm.

### EPR experiments

Individual EPR samples were prepared in a Vacuum Atmospheres glove box filled with Ar and operated at <3 ppm O_2_, and flash-frozen in liquid nitrogen before analysis. The reduced samples contained 10% (vol/vol) glycerol, 250 mM imidazole, 2 mM Na_2_S_2_O_4_ and 25 mM Tris‒HCl (pH 8.0), and the oxidized samples were prepared by incubating the reduced samples with excess indigo disulfonate (IDS) for 5 min. The sample concentration was 20 mg ml^−1^. EPR data were acquired using an ESP 300E spectrophotometer (Bruker) interfaced with an ESR-9002 liquid-helium continuous-flow cryostat (Oxford Instruments), with a microwave power of 5 mW, gain of 5 × 10^4^, modulation frequency of 100 kHz and modulation amplitude of 5 G. Eight scans of perpendicular-mode EPR spectra were recorded at 10 K (for each reduced sample) and at 15 K (for each oxidized sample), using a microwave frequency of 9.62 GHz.

### Cryo-EM grid freezing

Protein samples were diluted to 0.75 mg ml^−1^ in buffer containing 50 mM Tris‒HCl (pH 8.0), 500 mM NaCl and 1% glycerol in an anaerobic chamber. The sample (3 μl) was applied to Quantifoil R1.2/1.3 300 mesh Cu or Au ultrathin carbon grids that were negatively glow-discharged using a PELCO easiGlow set-up (Ted Pella). The grids were blotted for 2–6 s with a blot force of 1 using a Vitrobot Mark IV instrument (Thermo Scientific) set to 4 °C and 100% humidity in an anaerobic chamber. Grids were plunge-frozen in liquid ethane and stored in liquid nitrogen.

### Cryo-EM data collection

Grids were screened using a 200-keV Talos Arctica or Glacios system (Thermo Fisher Scientific) to assess ice thickness, particle quality and distribution. All movies were collected with a 300-keV FEI Titan Krios microscope equipped with a Thermo Fisher Scientific Selectris X energy filter and a Falcon 4i direct electron detector. Movies were collected using SerialEM at a pixel size of 0.743 Å pixel^−1^ with a total dose of 60 e^−^/Å^2^ spread over 1,404, 1,341 and 1,440 total frames for datasets 1, 2 and 3, respectively. The full data collection parameters are provided in Supplementary Table 1.

### Cryo-EM data processing

The cryo-EM processing pipeline is illustrated in Supplementary Fig. 2. CryoSPARC 4.2.1^42^ was used to process 4,641 movies with patch motion correction^43^ and patch contrast transfer function (CTF) estimation. A selection of 4,608 micrographs were subjected to blob-picking using circular and elliptical templates, resulting in 3,997,718 extracted particles. Multiple rounds of 2D classification resulted in a subset of 93,132 particles that were used for an ab initio 3D reconstruction. Further 2D classification was performed to increase the number of particles to 96,802 for non-uniform refinement of that initial model, resulting in a 4.42-Å *C*2-symmetric reconstruction. Additional particles were selected from 2D classification, and non-uniform refinement was repeated with 139,113 particles, resulting in a 4.24-Å *C*2-symmetric reconstruction.

An additional 5,415 movies were collected, of which 4,671 were template-picked, resulting in 3,531,405 extracted particles. The picked particles were parsed using heterogenous refinement between two classes, the aforementioned 4.24-Å refined map from the previous dataset and a lower-quality ab initio 3D reconstruction from undesirable particles. The particles sorted into the refined map class underwent several rounds of 2D classification until 82,899 particles remained. The 96,802 particles from the previous dataset were combined with this particle set to generate an ab initio 3D reconstruction containing a total of 179,701 particles. The two datasets were then combined, and multiple rounds of 2D classification were performed to select additional particles for refinement. Non-uniform refinement with 369,285 particles generated a 3.90-Å *C*1-symmetric reconstruction. This refined map then underwent 3D classification into six classes, resulting in the identification of an apo-NifEN class composed of 147,896 particles and a holo-NifEN class composed of 71,643 particles. Both classes were subjected to non-uniform refinement, resulting in a 3.84-Å *C*1-symmetric apo-NifEN reconstruction and a 4.22-Å *C*1-symmetric holo-NifEN reconstruction. The particles from five of the 3D classes were selected for non-uniform refinement using the apo-NifEN class as an initial model, which resulted in a 3.67-Å *C*2-symmetric reconstruction.

The final dataset was composed of 6,480 movies, of which 4,686 were selected for blob-picking using circular and elliptical templates, resulting in 1,471,180 extracted particles. Similar to the second dataset, the particles were parsed using heterogeneous refinement between the refined 3.67-Å map and a lower-quality ab initio 3D reconstruction. The particles sorted into the refined map class underwent multiple rounds of 2D classification for a total of 289,251 selected particles. These particles generated a 4.11-Å *C*1-symmetric non-uniform refined reconstruction, which was then used for 3D classification into six classes. Once more, individual classes for apo-NifEN and holo-NifEN were identified, and combined with the previous datasets.

The apo-NifEN classes from all three datasets were combined for a total of 246,031 particles, resulting in a *C*1-symmetric non-uniform refined reconstruction at 3.75 Å. The particles underwent another round of 3D classification into six classes to parse out the highest-resolution particles. This resulted in a class of 43,544 particles, which were used to generate a new ab initio 3D reconstruction for non-uniform refinement with additional particles, resulting in a 3.76-Å *C*1-symmetric reconstruction containing 126,645 particles. A mask was created around the well-resolved αβ_2_ trimer and used for particle subtraction of the unresolved α-subunit. The resulting 126,644 subtracted particles were used for local refinement and produced the final apo-NifEN reconstruction (EMD-70642) at a resolution of 3.72 Å.

Similarly, the holo-NifEN classes from all three datasets were combined for a total of 117,750 particles, resulting in a *C*1-symmetric non-uniform refined reconstruction at 3.94 Å. The particles underwent another round of 3D classification into three classes to parse out the highest-resolution particles. This resulted in a class of 40,671 particles, which were used to generate a new ab initio 3D reconstruction for non-uniform refinement, resulting in a 3.71-Å *C*1-symmetric reconstruction. A mask was created around the well-resolved αβ_2_ trimer and used for particle subtraction of the unresolved α-subunit. The resulting 40,670 subtracted particles were used for local refinement and produced the final holo-NifEN^in^ reconstruction (EMD-70643) at a resolution of 3.62 Å.

### Model building, refinement and validation

Atomic models for apo-NifEN and holo-NifEN^in^ were generated using the AlphaFold Protein Structure Database (AFDB)^44–46^ as the initial models. For holo-NifEN^in^, ligands SF4 and S5Q were used to model the O-cluster and L-cluster, respectively. The AlphaFold models and ligands were rigid body fit, combined with ChimeraX^47^ and positioned to best fit the cryo-EM density map using Coot 0.9.8.95^48^. The models were refined into the final corresponding maps using Phenix real-space refinement v1.21.2-5419^49,50^. Model validation was performed using Phenix and MolProbity^51^. Statistics are available in Supplementary Table 1. All figures were prepared with ChimeraX^47^, PyMOL^52^ and Microsoft PowerPoint. Supplementary Videos were prepared with ChimeraX^47^ and PyMOL^52^.

### AlphaFold 3 modelling

AlphaFold 3 modelling was carried out using the AlphaFold server (https://alphafoldserver.com/)^34^ and the sequences of the *nif*-encoded proteins of *A. vinelandii*. In the case of NifEN-B′ fusion, two copies of the NifE sequence and two copies of the NifN-NifB′ fusion sequence were used for modelling (Supplementary Fig. 12 presents the protein sequences). In the case of the NifEN/NifH complex, two copies of the NifE sequence and two copies of the NifN sequence were used along with four copies of the NifH sequence for modelling (Gene ID 643803062; Locus Tag Avin_01380; https://img.jgi.doe.gov/).

### Negative-stain EM

All samples were subjected to negative stain EM analysis as follows^53^. Each sample, at concentrations of 0.075 or 0.1 mg ml^−1^, was pipetted (3 µl) onto Formvar/carbon-coated 400-mesh Cu grids (Ted Pella). All grids were first negatively glow-discharged using a PELCO easiGlow set-up (Ted Pella) before the addition of protein. Excess sample was removed with filter paper and the grids washed three times with 50-µl Milli-Q water drops. The grid was stained quickly with a 50-µl drop of freshly prepared 0.75% uranyl formate (Electron Microscopy Sciences) and stained again for 20 s on a second 50-µl drop of stain. Finally, the grid was dried in vacuum. All grids were imaged using a JEOL JEM-2100F TEM system equipped with a Gatan OneView (4k × 4k) camera. Micrographs were contrast-enhanced using Fiji^54^.

Drift-corrected images were converted from dm4 to mrc files using dm2mrc, a program of IMOD^55^, and imported into cryoSPARC 4.7.0^42^ with a pixel size of 1.14678 Å pixel^−1^. Images were patch CTF-corrected and subjected to blob-picking and template-picking. Particles were extracted at 320 pixels and underwent 2D classification with a minimum separation distance of 85 Å. The particles of specific 2D classes were selected for ab initio 3D generation and subsequent non-uniform refinement. The AlphaFold 3-predicted models of the NifEN-B′ fusion and the NifEN/NifH complex were converted into 25-Å lowpass-filtered 3D reconstructions using ChimeraX^47^. The AlphaFold and negative-stain 3D reconstructions were aligned using ChimeraX^47^ and resampled to produce 2D projections using the create templates job.

### Design of NifEN variants

The candidates for the NifB–NifN interface mutants were chosen by visual inspection of the interface between NifN and NifB′ in the AlphaFold 3 model of the NifEN-B′ fusion, and through interface analysis by PDBePISA^56^ (https://www.ebi.ac.uk/pdbe/pisa/). The NifEN tunnel variants were selected by examining residues lining the proposed L-cluster trafficking pathway, with particular emphasis on arginines. These residues were targeted to disrupt electrostatic and hydrogen-bonding interactions with the L-cluster, thereby hindering its transport. In addition, mutations of the known and putative L-cluster ligands, Cys15 and Cys25, located at the front opening of the tunnel, were included to further impede L-cluster maturation.

### Reporting Summary

Further information on research design is available in the Nature Portfolio Reporting Summary linked to this Article.

## Supplementary information

Supplementary Information Supplementary Note 1, Figs. 1–20 and Table 1.

Reporting Summary

Supplementary Video 1 Apo-NifEN to holo-NifEN^in^volume morph. The six classes from the 3D classification of dataset 3 were morphed in order from apo-NifEN to holo-NifEN^in^ using ChimeraX^47^to illustrate the structural heterogeneity of the α-subunit binding the L-cluster.

Supplementary Video 2 Apo-NifEN to holo-NifEN^in^ model morph. The structural models shown in Supplementary Fig. 8a were morphed using PyMOL^52^. Seventy-five output states with three refinement cycles were calculated. The hypothetical movement of the L-cluster during its incorporation into NifEN is rendered, ending at the position of the L-cluster in the holo-NifEN^in^ structure.

Supplementary Video 3 Holo-NifEN^in^ to holo-NifEN^out^ model morph. The structural models shown in Supplementary Fig. 8b were morphed using PyMOL^52^. Seventy-five output states with three refinement cycles were calculated.

Supplementary Video 4 L-cluster trafficking from apo- to holo-NifEN^out^. Supplementary Videos 2 and 3 were combined sequentially to illustrate the movement of the L-cluster during the two-step conversion from apo-NifEN, via holo-NifEN^in^, to holo-NifEN^out^.

Supplementary Video 5 Apo-NifDK to holo-NifDK model morph. The structural models shown in Supplementary Fig. 8c (left) were morphed using PyMOL^52^. Seventy-five output states with three refinement cycles were calculated. The hypothetical movement of the M-cluster during its incorporation into NifDK is rendered, ending at the position of the M-cluster in the holo-NifDK structure.

## Data Availability

The negative-stain maps and cryo-EM maps associated with this study have been deposited to the Electron Microscopy Data Bank (EMDB) under accession codes EMD-73406 (negative-stained *A. vinelandii* NifEN-B′ fusion), EMD-73407 (negative-stained *A. vinelandii* NifEN/NifH ADP·AIF_4_^−^-stabilized complex), EMD-70642 (L-cluster free apo-NifEN expressed in *E. coli*) and EMD-70643 (L-cluster inward-bound holo-NifEN expressed in *E. coli*). The atomic coordinates associated with this study have been deposited to the Protein Data Bank (PDB) under accession codes 9ONJ (L-cluster free apo-NifEN expressed in *E. coli*) and 9ONK (L-cluster inward-bound holo-NifEN expressed in *E. coli*). Previously reported models referred to in this paper can be found under accession codes 1L5H (FeMo-cofactor-deficient nitrogenase MoFe protein), 1N2C (nitrogenase complex from *A. vinelandii* stabilized by ADP-tetrafluoroaluminate), 3PDI (precursor-bound NifEN), 3U7Q (*A. vinelandii* nitrogenase MoFe protein at atomic resolution) and 7JMB (crystal structure of nitrogenase iron-molybdenum cofactor biosynthesis enzyme NifB from *Methanothermobacter thermautotrophicus* with three Fe_4_S_4_ clusters). All other data are available from the authors upon reasonable request.
