# Supplementary material for: Structural insights into metallocluster trafficking in the nitrogenase assembly scaffold NifEN
Source: Nat Catal. 2026 Mar 3;9(3):281–94. doi: 10.1038/s41929-026-01489-9 (PMC13031127; doi:10.1038/s41929-026-01489-9)
Supplement: Supplementary file 1 — Supplementary Note 1, Figs. 1–20 and Table 1. [file 41929_2026_1489_MOESM1_ESM.pdf]

nature catalysis

Supplementary information https://doi.org/10.1038/s41929-026-01489-9

Structural insights into metallocluster trafficking in the nitrogenase assembly scaffold NifEN

In the format provided by the authors and unedited

Supplementary Information

Table of Contents

Supplementary Notes…..2
  Supplementary Note 1.. 2
Supplementary Figures... 3
  Supplementary Figure 1..3
  Supplementary Figure 2..5
  Supplementary Figure 3..6
  Supplementary Figure 4..7
  Supplementary Figure 5..8
  Supplementary Figure 6..9
  Supplementary Figure 7..10
  Supplementary Figure 8..11
  Supplementary Figure 9..12
  Supplementary Figure 10..13
  Supplementary Figure 11..14
  Supplementary Figure 12..15
  Supplementary Figure 13..16
  Supplementary Figure 14..18
  Supplementary Figure 15..20
  Supplementary Figure 16..21
  Supplementary Figure 17..22
  Supplementary Figure 18..23
  Supplementary Figure 19..24
  Supplementary Figure 20..25
Supplementary Tables...26
  Supplementary Table 1.. 26

Supplementary Notes

Supplementary Note 1:

While the overall map resolutions are moderate, many regions achieve high local resolution, allowing confident placement of metal clusters and side chains, including those of the flexible residues, such as arginines, near the clusters. The angular distribution plots for both maps reveal distinct hot spots where particles are well aligned to NifN (Supplementary Fig. 3a), indicating some preferred orientations. However, this bias is modest and does not compromise map quality. The apo-NifEN dataset displays a broader distribution with areas of oversampling, whereas the holo-NifEN^{in} dataset exhibits a cleaner and more balanced distribution, consistent with improved particle alignment and higher-quality reconstructions.

2D classifications further support these observations, showing multiple orientations of NifEN with a range of particle views contributing to the final maps. The highest-resolution information arises predominantly from specific orientations, yet sufficient angular coverage across other views ensures accurate overall reconstructions. Areas of lower local resolution correlate with regions of increased structural flexibility, particularly where metal clusters are absent, and these dynamics are evident even within individual 2D classes.

Overall, the NifN models are consistent between apo and holo forms, with most conformational variability localized to the α domains surrounding the clusters. Imposing C2 symmetry reduced the resolution of NifE, suggesting that structural asymmetry and dynamic variation in these domains contribute to the observed differences between the two states.

Supplementary Figures

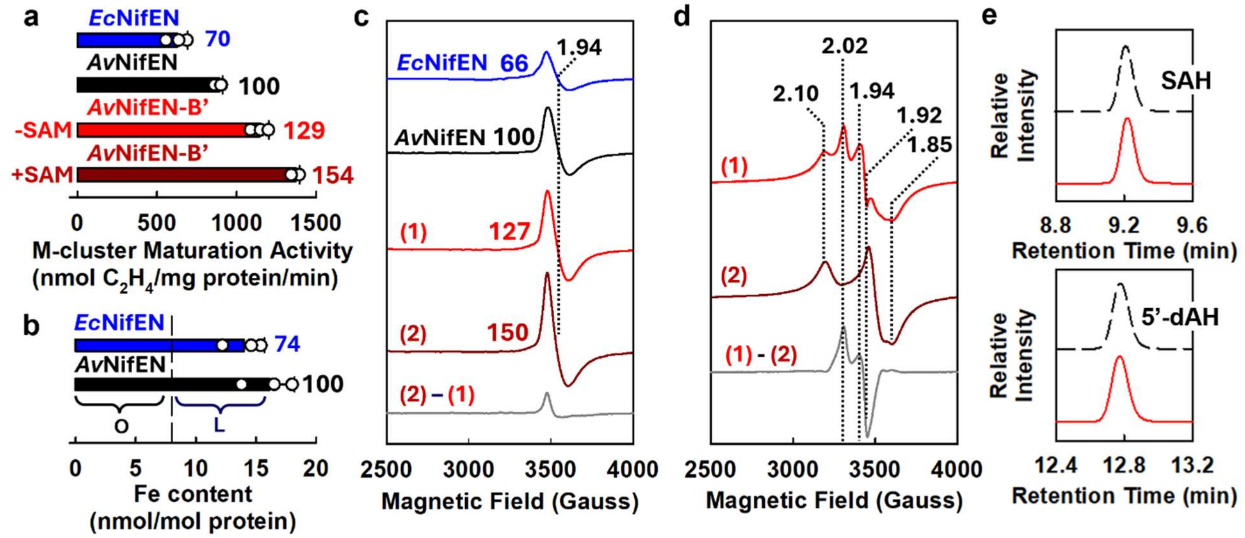

Supplementary Fig. 1 | Biochemical and spectroscopic characterization of NifEN species used in this study. a, M-cluster maturation assays (*n*=3) of *A. vinelandii* NifEN heterologously expressed in *E. coli* (*Ec*NifEN, blue) or homologously expressed in the native host (*Av*NifEN, black), and *A. vinelandii* NifEN-B' fusion homologously expressed in the native host (*Av*NifEN-B') without (red) or with (brown) the addition of *S*-adenosylmethionine (SAM). The activities are shown in bold font and expressed as percentages, with the activities of *Av*NifEN set as 100%. Note that the activity of wildtype NifDK of *A. vinelandii* is ~2000 nmol C_2H_4/mg protein/min[7,8]. However, the standard nitrogenase assay (involving NifH and NifDK) differs fundamentally from the cofactor maturation assay (involving various biosynthetic components in addition to NifH and NifDK) and, therefore, activity values are most meaningful when compared within, rather than between, each assay category. Data are presented as mean values ± SD. **b,** Fe contents of *Ec*NifEN and *Av*NifEN (*n*=3). The L-cluster content of *Ec*NifEN (74%, blue), as compared to that of *Av*NifEN (100%, black), was calculated by subtracting 8 Fe atoms in the two permanent O-clusters ([Fe_4S_4]) from the total Fe content per NifEN tetramer (*Ec*NifEN, 14±1.7 total Fe/mol protein; *Av*NifEN, 16.1±2.1 mol total Fe/mol protein). Data are presented as mean values ± SD. **c,** Perpendicular-mode EPR spectra of IDS-oxidized *Ec*NifEN (blue), *Av*NifEN (black), and *Av*NifEN-B' without ((1), red) and with ((2), brown) incubation with SAM, showing the L-cluster-specific signal at *g*=1.94. The intensities of the L-cluster-specific signals are shown in bold font and expressed as percentages, with the signal intensity of *Av*NifEN set as 100%. Compared to *Av*NifEN, the relative M-cluster maturation activity (a, 70%), L-cluster content (**b**, 74%) and L-cluster-specific EPR signal intensity (**c**, 66%) of *Ec*NifEN align well with one another. Note that the magnitude of the L-cluster-specific signal of *Av*NifEN-B' increases upon incubation with SAM (**c**, (2) *vs.* (1)), which results from conversion of the extra K-clusters to L-clusters on the *Av*NifB' entity of *Av*NifEN-B'. Thus, in the *IDS-oxidized* state, the L-clusters derived from the extra K-clusters give rise to the difference spectrum of *Av*NifEN-B' before and after incubation with SAM (**c**, (2)–(1)). **d,** Perpendicular-mode EPR spectra of dithionite-reduced *Av*NifEN-B' without (**d**,

(1)) and with (**d**, (2)) incubation with SAM. The *g* values are indicated. Note that in the absence of SAM (**d**, (1)), *Av*NifEN-B' displays a composite spectrum comprising the *S*=1/2 signals of the K-, L-, and O-clusters; whereas in the presence of SAM (**d**, (2)), *Av*NifEN-B' loses the K-cluster-associated feature upon SAM-dependent K- to L-cluster transformation. Thus, in the *dithionite-reduced* state, the K-cluster specific, *S*=1/2 signal can be derived from the difference spectrum of *Av*NifEN-B' before and after incubation with SAM (**d**, (1)–(2)). (**e**) HPLC-analysis of SAM-cleavage products of *Av*NifEN-B' (solid red) as compared to standards (dashed gray), showing that *Av*NifEN-B' is fully functional in cleaving SAM into SAH (upper) and 5'-dAH (lower).

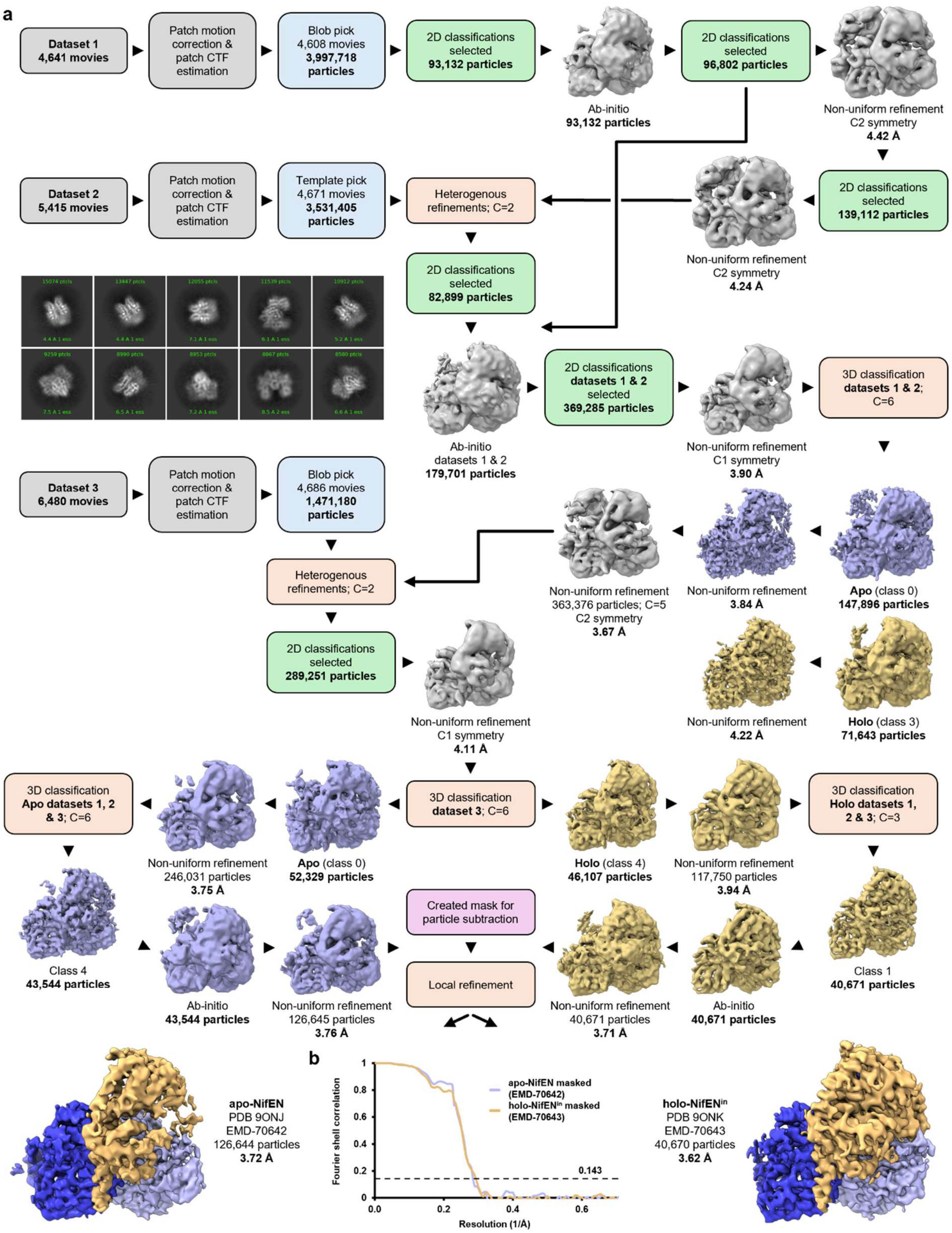

Supplementary Fig. 2 | Cryo-EM processing flowchart of NifEN using cryoSPARC. a, Processing workflow resulting in apo-NifEN and holo-NifEN^{in} reconstructions. **b,** Fourier shell correlation (FSC) plot of the two final reconstructions. FSC = 0.143 is indicated by a dashed black line.

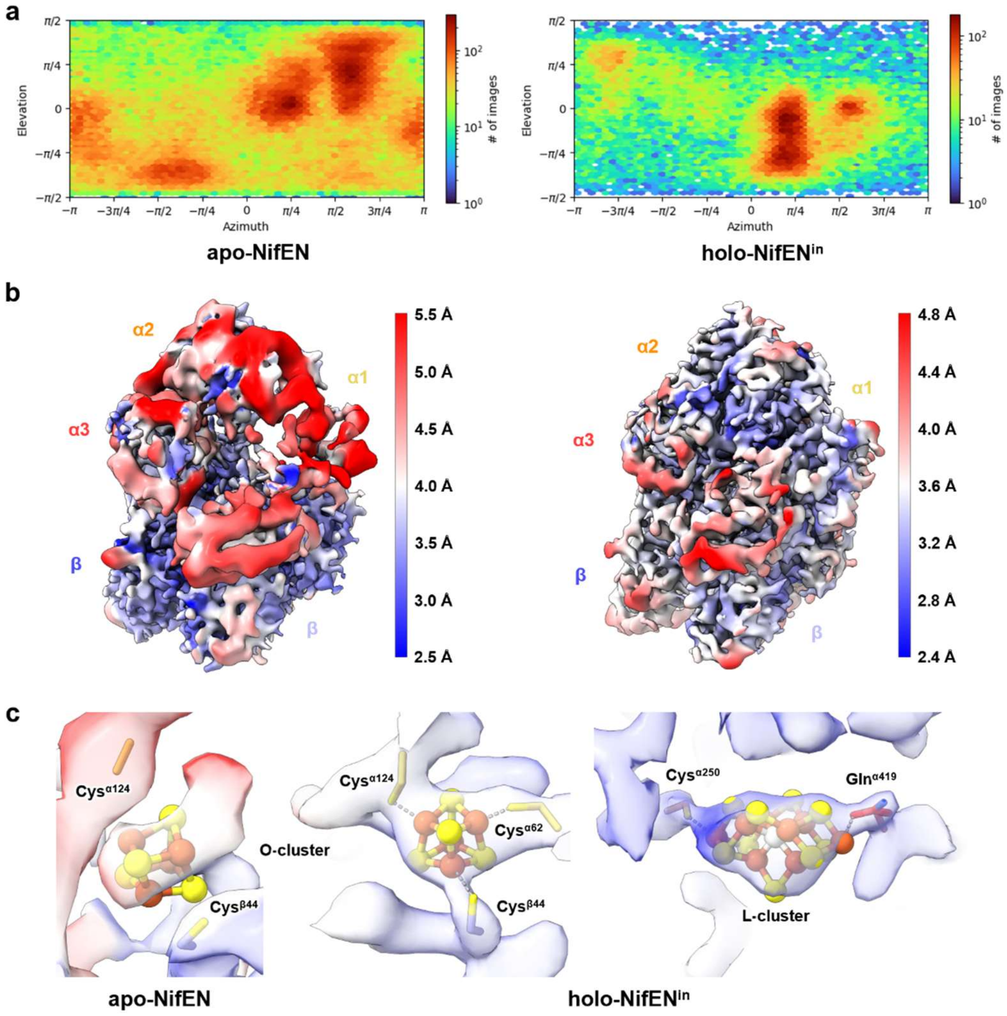

Supplementary Fig. 3 | Local resolution of apo-NifEN and holo-NifEN^{in}. a, Angular distribution plot of particles in the final apo-NifEN (left) and holo-NifEN^{in} (right) reconstructions. **b,** Local resolution of apo-NifEN (left) and holo-NifEN^{in} (right) calculated within cryoSPARC. **c**, Close-up views of the O-cluster (left, center) and the L-cluster (right), overlaid with local resolution. The predicted location of the O-cluster in apo-NifEN (left) was determined by superimposition with the holo-NifEN^{in} structure. Both clusters are shown in ball-and-stick representation, with side chains of the ligands and the nearby hydrogen-bonding residues shown in stick presentation.

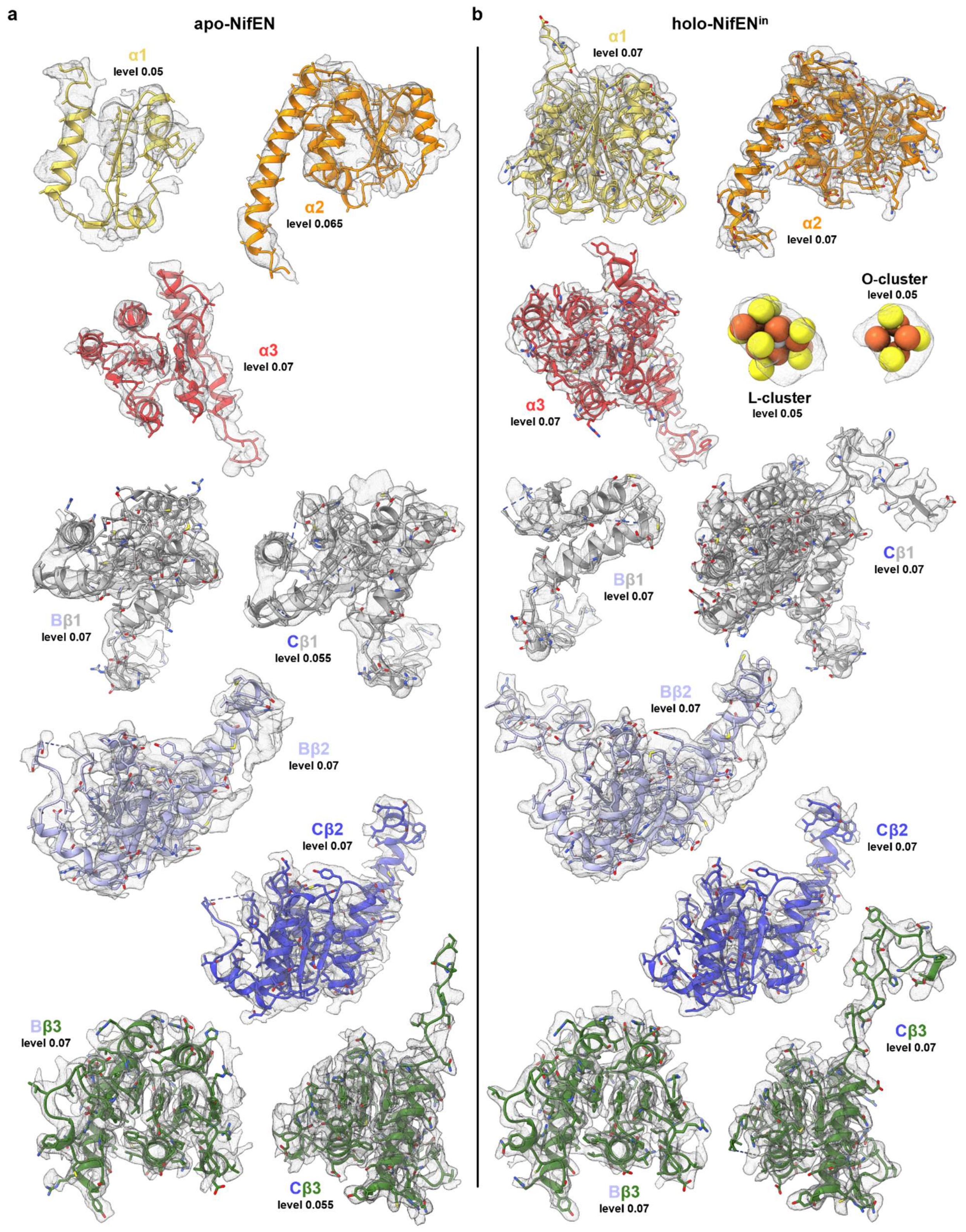

Supplementary Fig. 4 | Map-to-model fits of apo-NifEN and holo-NifEN^{in}. a,b, Domains of (**a**) apo-NifEN and (**b**) holo-NifEN^{in} fitted into EMD-70642 and EMD-70643, respectively. The threshold level of the map density is labeled for each domain.

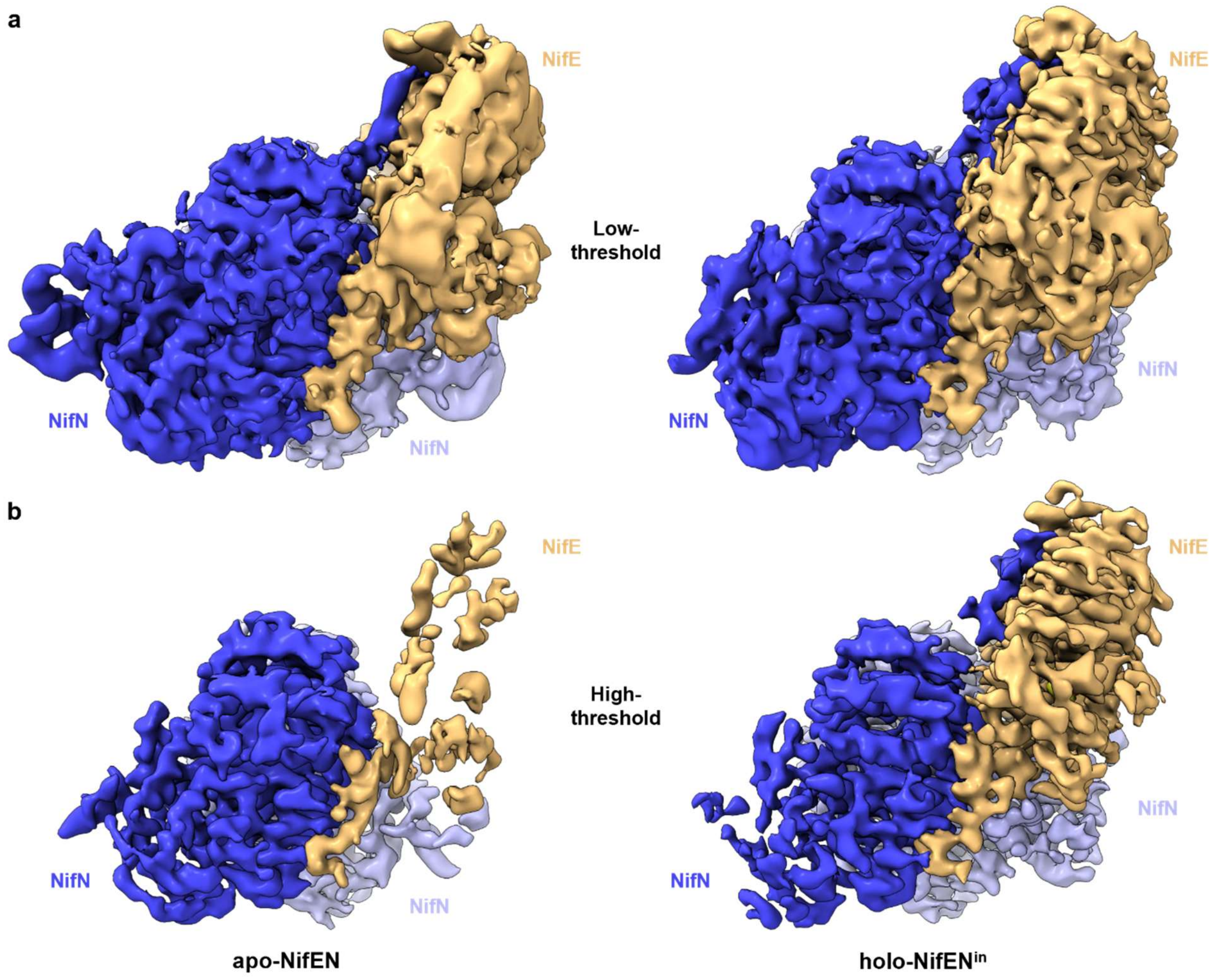

Supplementary Fig. 5 | High- and low-threshold maps of apo-NifEN and holo-NifEN^{in}. a,b, Low-threshold (**a**) and high-threshold (**b**) of the apo-NifEN (left) and holo-NifEN^{in} (right) density maps highlighting the absence of high-resolution features in the NifE (α) subunit of apo-NifEN.

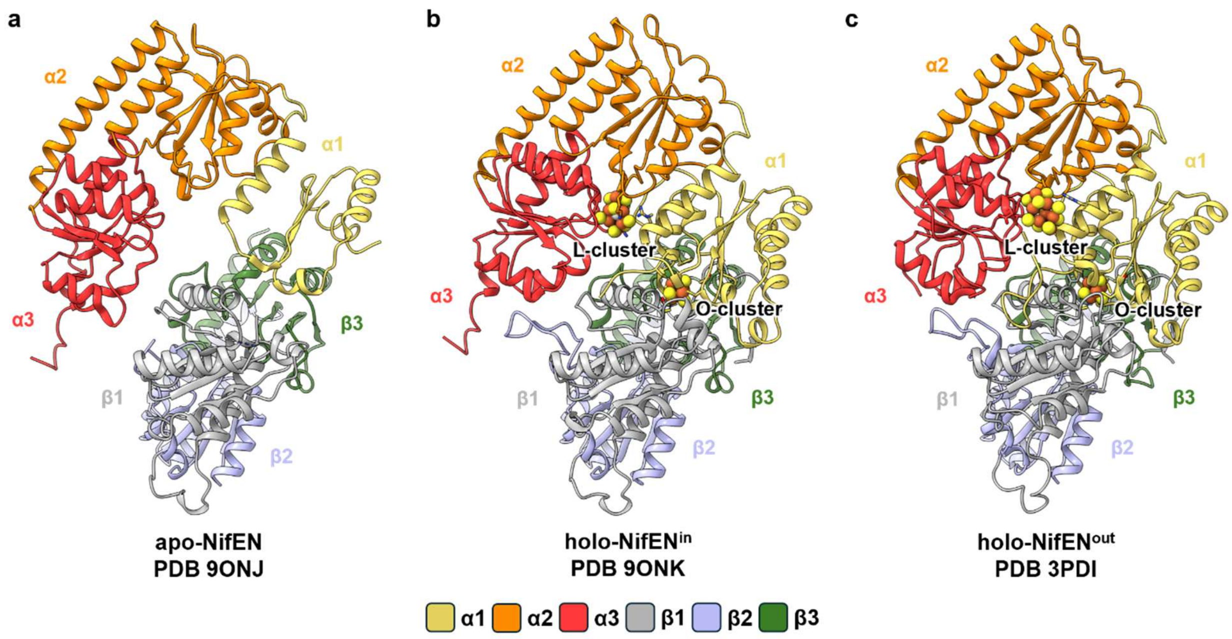

Supplementary Fig. 6 | Visualization of NifEN domains. a-c, Structures of the αβ-pairs of (**a**) apo-NifEN, (**b**) holo-NifEN^{in}, and (**c**) holo-NifEN^{out}. The three domains of the NifE (α)- and NifN (β)-subunits are colored as indicated in the figure. The figure was generated with PDB entries 3PDI[26], 9ONJ and 9ONK.

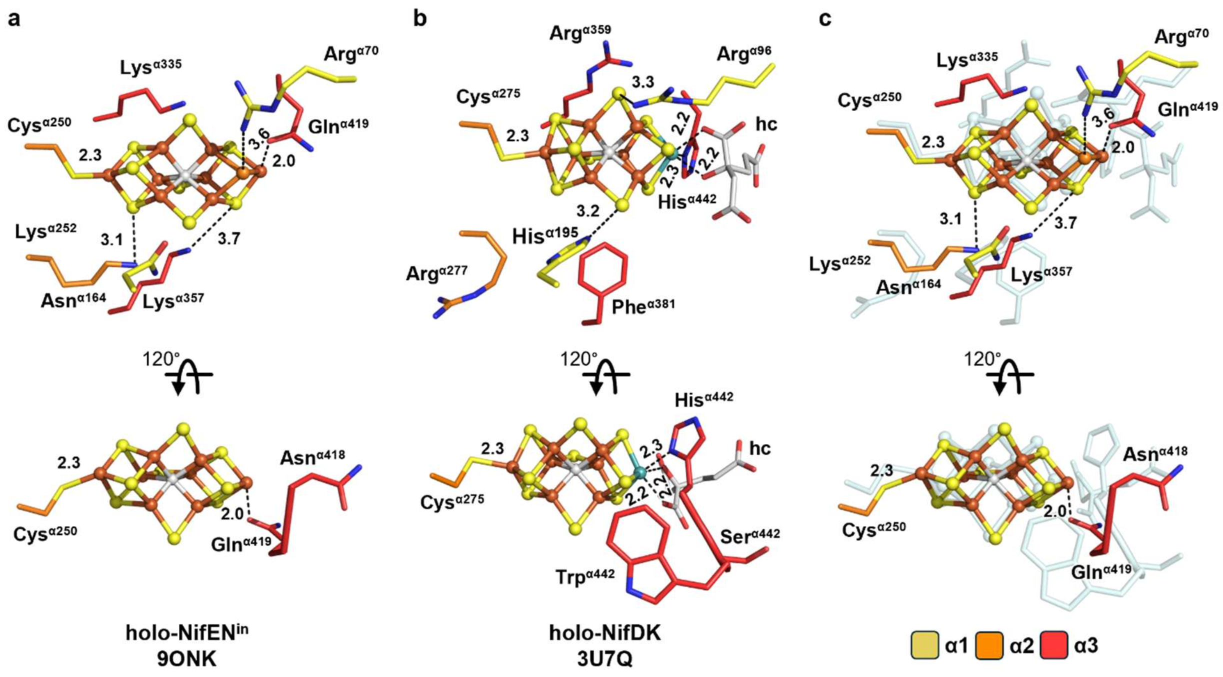

Supplementary Fig. 7 | Interactions between residues and clusters in holo-NifEN^{in} and holo-NifDK. a-c, Detailed views (shown in two perspectives) of the protein environment surrounding (**a**) the L-cluster in holo-NifEN^{in} and (**b**) the M-cluster in holo-NifDK, and (**c**) a superimposed depiction of the cluster environments in holo-NifEN^{in} and holo-NifDK. Shown are the side-chain residues located in close proximity to the clusters. The side chains are presented as sticks and colored according to the α-subunit domains they reside in. The clusters are depicted as ball-and-stick models, with atoms colored as follows: Fe, orange; S, yellow; O, red; C, gray; Mo, cyan. In the superimposed view (**c**), the M-cluster environment is rendered transparent cyan. The figure was prepared using PyMOL[52] with PDB entries 3U7Q[14] and 9ONK.

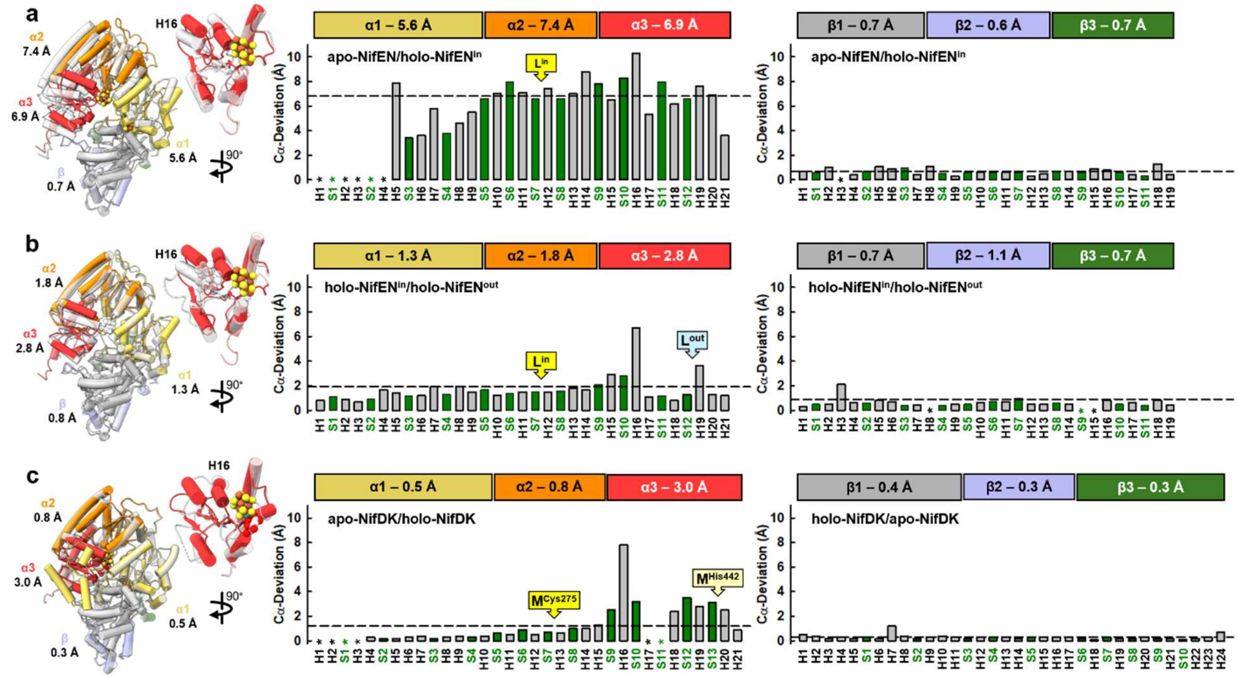

Supplementary Fig. 8 | Cα-deviations derived from an overlay of NifEN and NifDK. a-c, Overlaid αβ-pairs and α3-domains (left) and Cα-deviations of helices (H) and sheets (S) across the three domains of the α-subunit (middle) and the β-subunit (right) of (**a**) apo-NifEN *vs.* holo-NifEN^{in}, (**b**) holo-NifEN^{in} *vs.* holo-NifEN^{out}, and (**c**) apo-NifDK *vs.* holo-NifDK. In the superimposed structures (**a-c**, left), apo-NifEN, holo-NifEN^{out} and apo-NifDK are shown in gray and rendered in the background, and the three domains of the α- and β-subunits of holo-NifEN^{in} (**a**, **b**, left) and holo-NifDK (**c**, left) colored as those in Fig. 4. The average Cα-deviation for each domain or subunit is indicated next to the superimposed structures (left) or above the bar charts (middle, right). The dashed lines in the bar charts indicate the average Cα-deviations of the entire α- and β-subunits (middle, right). ChimeraX[47] was used to generate the superimposed structures and calculate the Cα-deviations of helices and sheets in the overlaid models based on PDB entries 9ONJ, 9ONK, 3PDI[26], 1L5H[27], and 3U7Q[14]. The locations of the ligands for the L-cluster ($\text{Cys}^{\alpha25}$ for L^{out}; $\text{Cys}^{\alpha250}$ and for $\text{Gln}^{\alpha419}$ L^{out}) and the M-cluster ($\text{Cys}^{\alpha275}$ and $\text{His}^{\alpha442}$) are indicated in the figure.

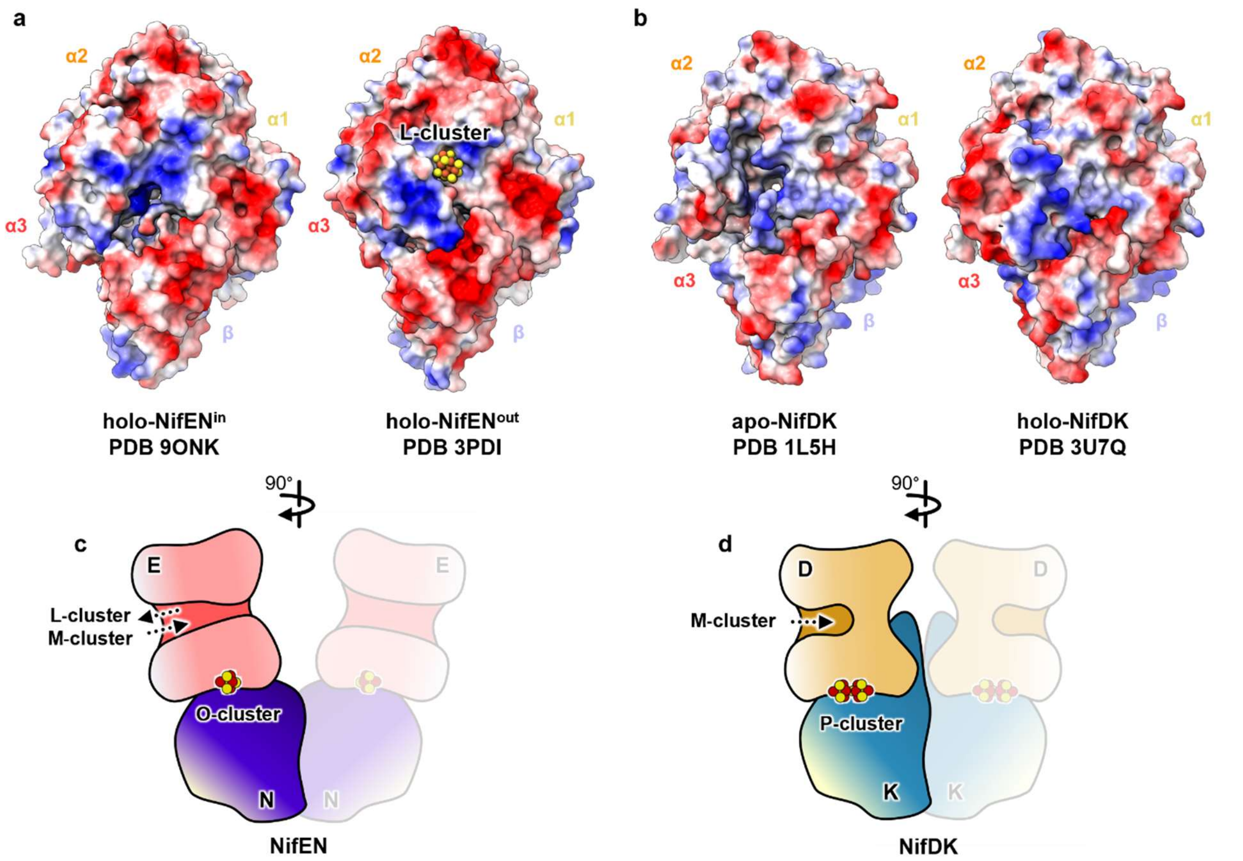

Supplementary Fig. 9 | Visualization of the surfaces of NifEN and NifDK. a,b, Electrostatic surface charge representations of (**a**) holo-NifEN^{in} and holo-NifEN^{out} and (**b**) apo-NifDK and holo-NifDK, with the negative and positive surface charges indicated in red and blue, respectively. The atoms of the surface-exposed L-cluster in holo-NifEN^{out} are depicted as red (Fe) and yellow (S) spheres. **c,d,** The proposed, analogous metal cluster trafficking pathways in NifEN (**c**) and NifDK (**d**) during the M-cluster maturation process are shown in schematic presentation. Upon interaction with NifH, the L-cluster on NifEN (**c**) is transferred from its internal site to an exposed surface location to undergo maturation into an M-cluster. This event is presumably followed by a transfer of the M-cluster to its internal binding site in NifEN (where the cofactor is better protected) prior to transfer of the M-cluster back to the surface of NifEN upon interaction with apo-NifDK. Once relayed from the surface of apo-NifEN to the surface of NifDK (**d**), the M-cluster travels through a pathway to its target binding site, resulting in the formation of a catalytically active holo-NifDK. The figure was generated with PDB entries 3PDI[26], 1L5H[27], and 3U7Q[14].

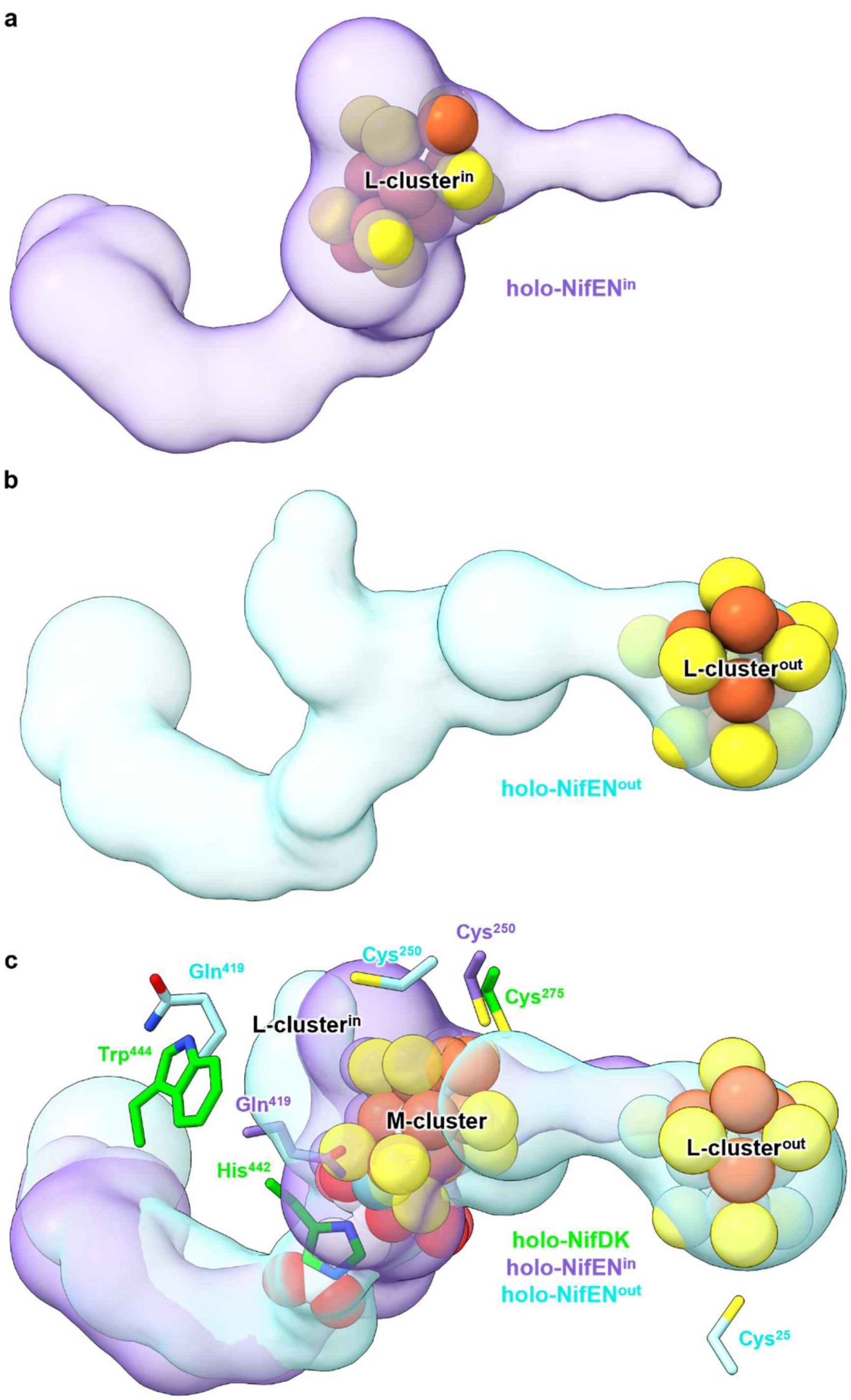

Supplementary Fig. 10 | Overlay of the clusters in holo-NifEN and holo-NifDK. a,b, Potential tunnels in (**a**) holo-NifEN^{in} (purple) and (**b**) holo-NifEN^{out} (cyan) encompassing the L-cluster. **c,** Superimposition of the tunnels identified for holo-NifEN^{in} (purple) and holo-NifEN^{out} (cyan) with metal clusters along the tunnel paths shown as ball-and-stick models. The position of the M-cluster was determined by superimposing holo-NifEN^{in} with the holo-NifDK structure (PDB 3U7Q). Shown are the side-chain residues located in close proximity to the clusters. Tunnel analysis was performed using MOLEonline and the figure was generated with PDB entries 3PDI[26], 3U7Q[14], and 9ONK.

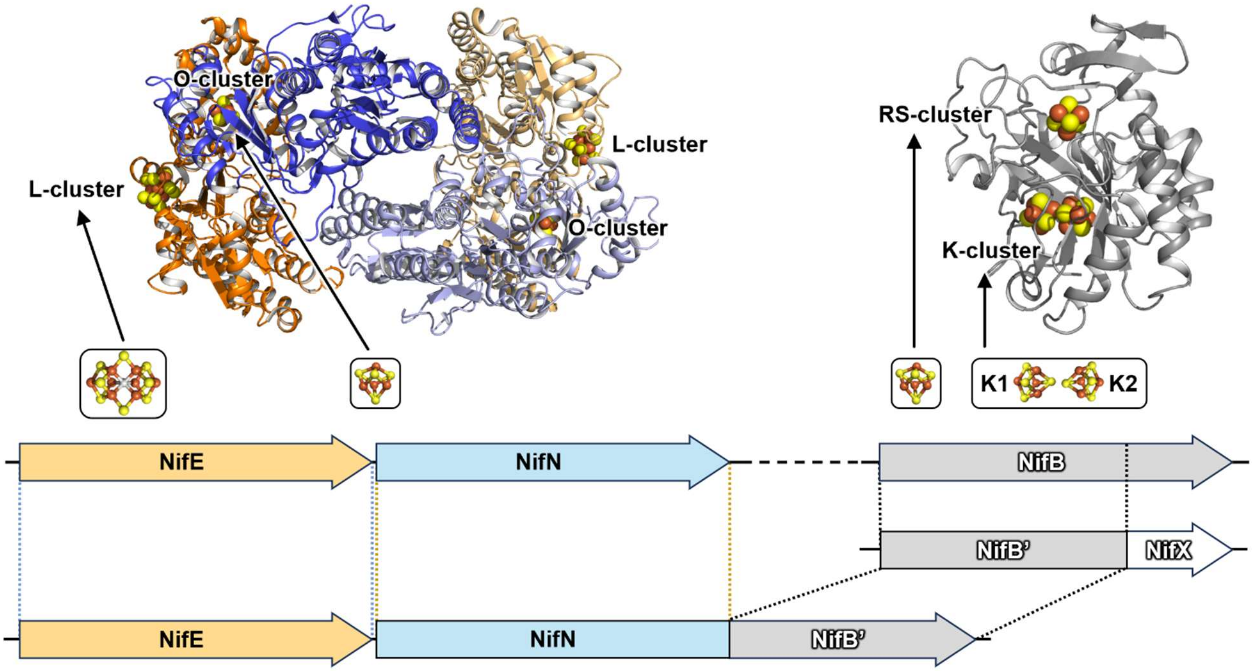

Supplementary Fig. 11 | Generation of the NifEN-B' fusion protein. Shown are the structures of holo-NifEN^{out} containing the L- and O-clusters (upper, left) and NifB containing the RS- and K-clusters (upper, right). The subunits of the tetrameric NifEN are colored as those in Fig. 1, and the monomeric NifB is shown in gray. The clusters are depicted and colored as those in Fig. 2. The C-terminus of *A. vinelandii* NifN was fused to the N-terminus of *A. vinelandii* NifB (lower panel). Additionally, the non-essential NifX domain of NifB was deleted (*see* Supplementary Fig. 12 for protein sequences). The figure was generated using PyMOL[52] with PDB entries 3PDI[26] and 7JMB[22].

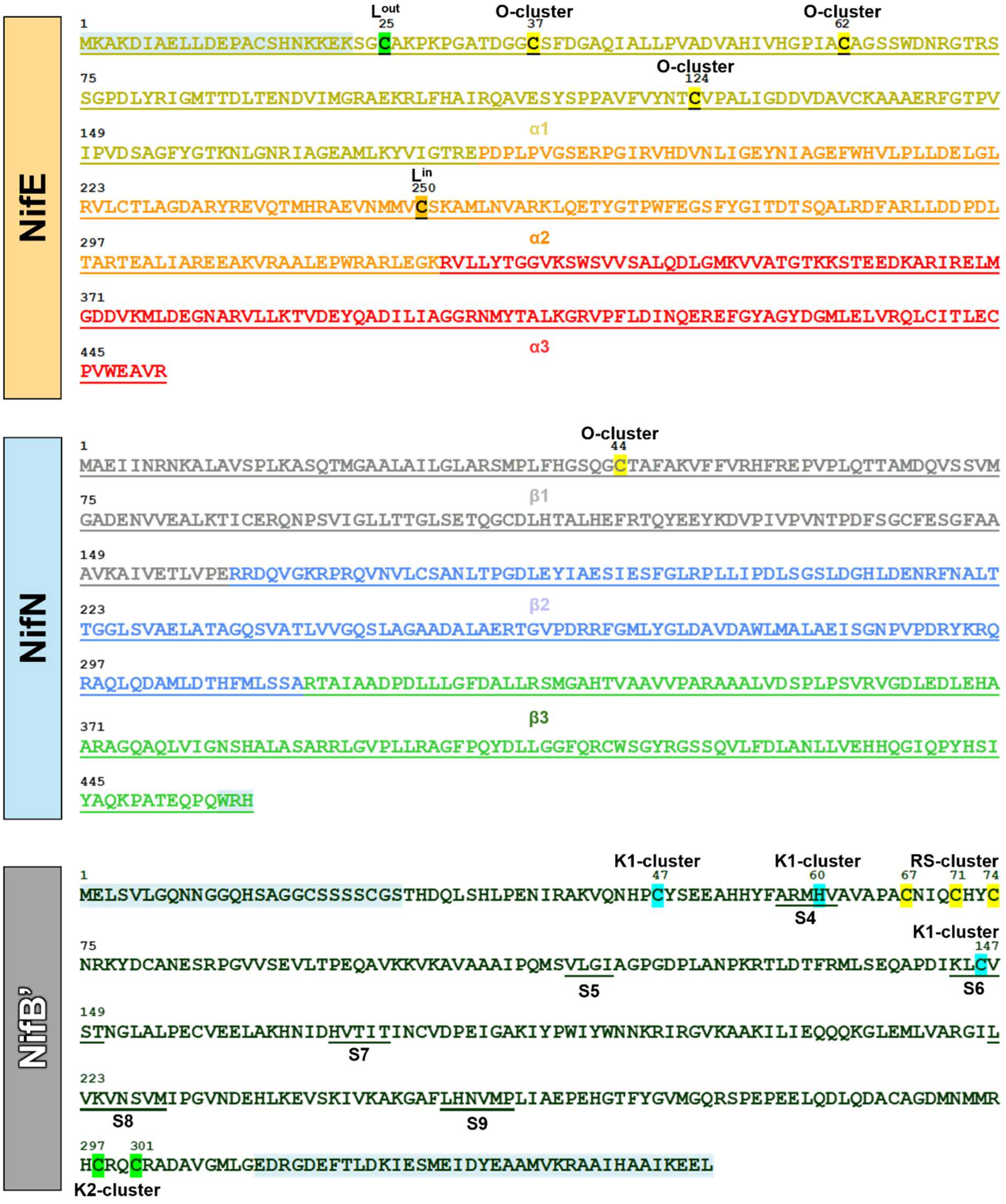

Supplementary Fig. 12 | Protein sequences of *A. vinelandii* NifEN and NifB'. The sequences corresponding to the three domains of NifE (α-subunit) and NifN (β-subunit) are colored as those in Supplementary Fig. 6. Shown are the ligands of the L-clusters (L^{in} and L^{out}) and the O-cluster of NifEN, and the ligands of the RS- and K-clusters (comprising K1- and K2-clusters) of NifB. The characteristic β-sheets (S) of the radical-SAM enzyme NifB are indicated. Disordered regions, highlighted in light blue, were removed for AlphaFold 3 analyses.

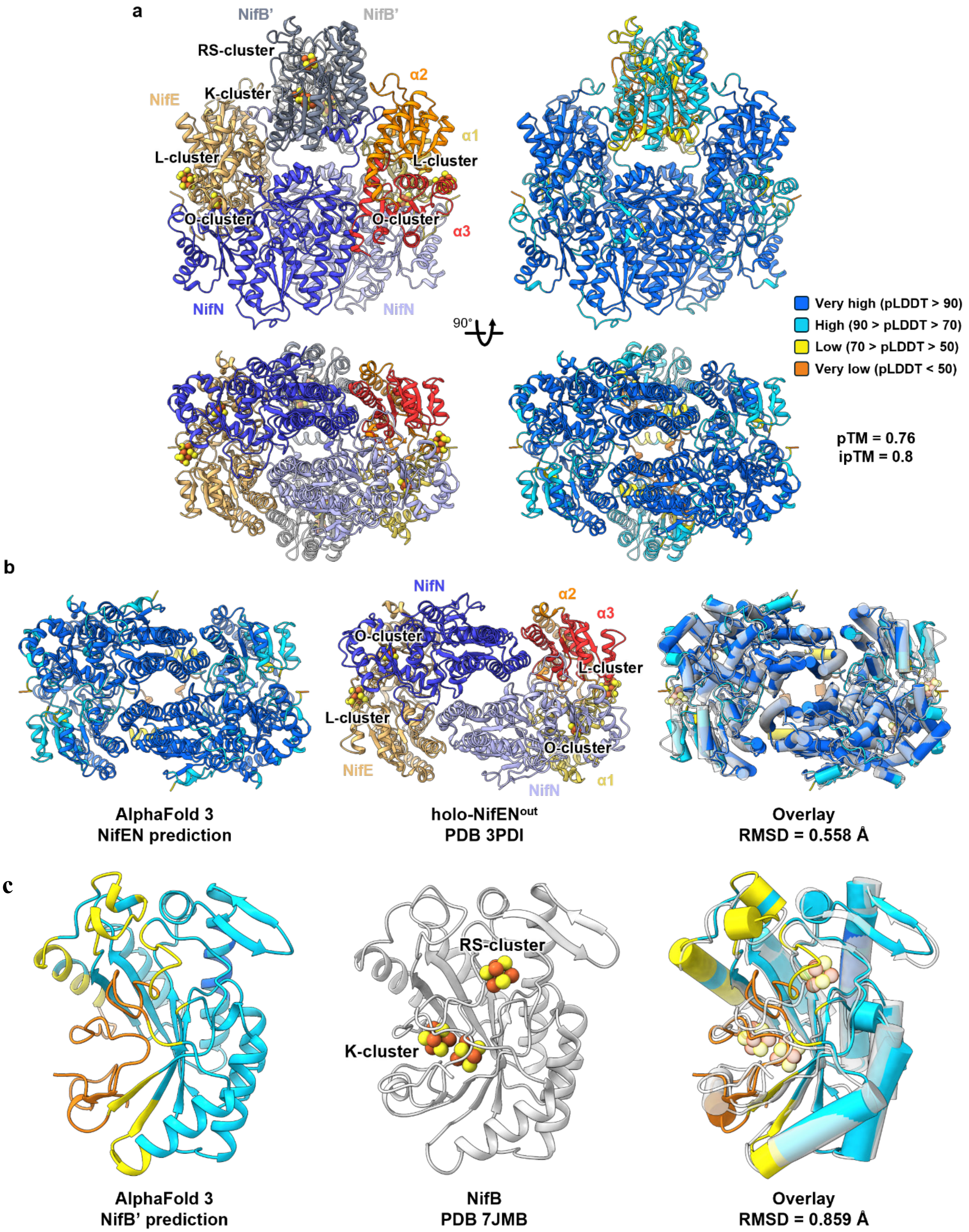

Supplementary Fig. 13 | Confidence metrics of the AlphaFold 3 predicted NifEN-NifB' fusion. a, Predicted structure of the NifEN-B' complex, shown with the molecular twofold axis aligned vertically (upper) and along the viewing direction (lower). Subunits and domains are

colored as those in Fig. 6 (left) or color-coded based on the prediction confidence as indicated in the figure (right). The overall pTM and ipTM scores of 0.76 and 0.8, respectively, reflect a high confidence of the prediction. The predicted locations of the L- and O-clusters in NifEN, and the RS- and K-clusters in NifB, are indicated by clusters in ball-and-stick presentation (left). **b,c,** The locations of the clusters were determined by superimposing (**b**) the NifEN portion of the model (left) with the structure of holo-NifEN^{out} (center, PDB 3PDI[26]) and (**c**) the NifB portion of the model (left) with the structure of NifB (center, PDB 7JMB[22]). Both superimpositions show RMSDs below 1 Å (0.558 Å for NifEN and 0.859 Å for NifB), underscoring the high quality of the model. The models (**b**, **c**, left) are shown with the same coloring as that in (**a**, right) and the structures (**b**, **c**, middle) are shown with the coloring as that in (**a**, left) except for the structures in the overlays (**b**, **c**, right), where transparency is applied.

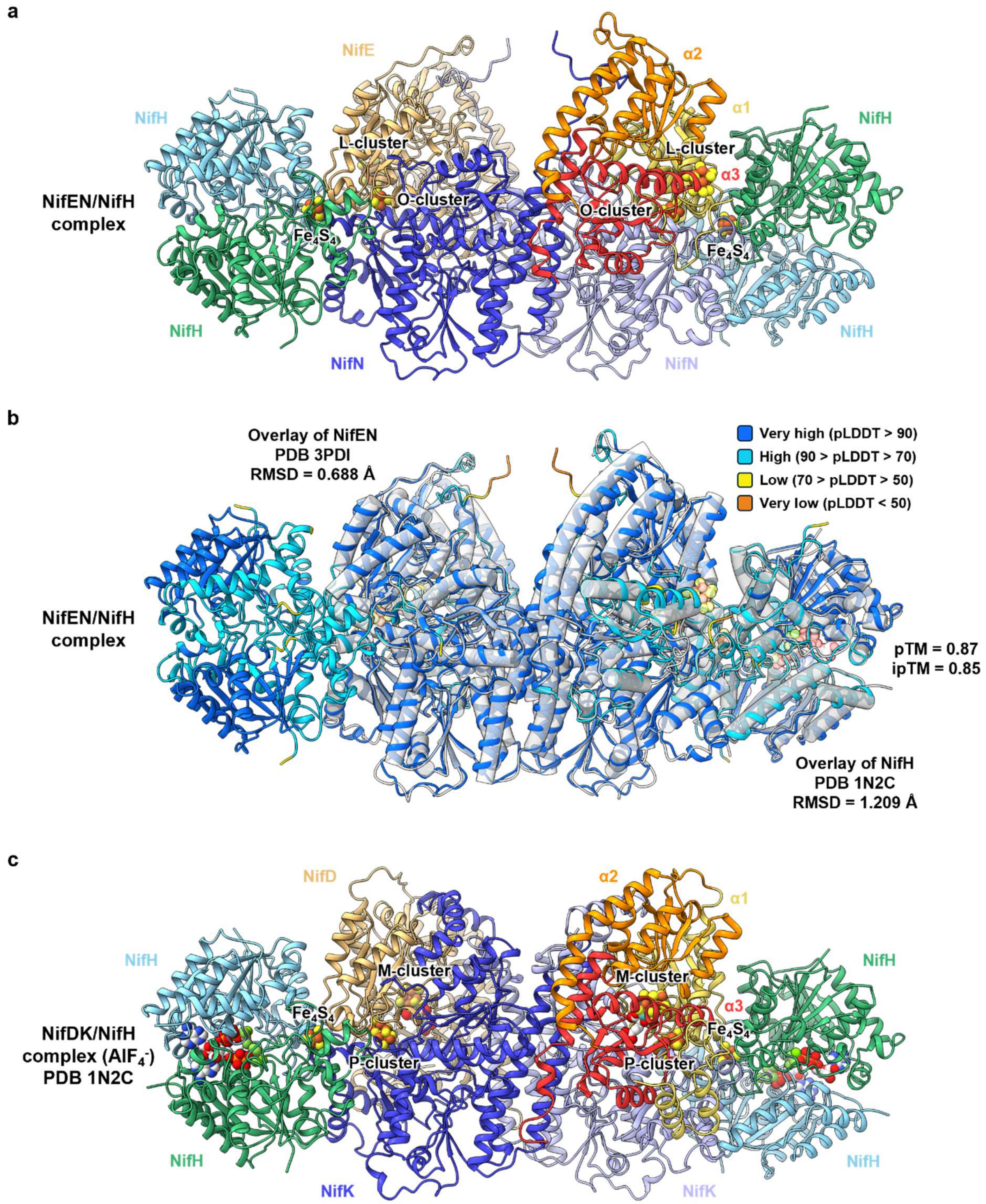

Supplementary Fig. 14 | Confidence metrics of the AlphaFold 3 predicted NifEN/NifH complex. a,b, Predicted structure of the NifEN/NifH complex with (**a**) subunits and domains colored as those in Fig. 6 and (**b**) color-coded based on the prediction confidence as indicated in the figure. The overall pTM and ipTM scores of 0.87 and 0.85, respectively, indicate a high confidence of the prediction. The predicted positions of the L- and O-clusters in NifEN and the

[Fe_4S_4] cluster in NifH are indicated by clusters in ball-and-stick presentation (**a**). The positions of these clusters were determined by superimposing the NifEN portion of the model with the holo-NifEN^{out} structure (PDB 3PDI[26]) and the NifH portion with the NifH component in the NifDK/NifH complex structure (PDB 1N2C[12]). Both superimpositions show low RMSD values (0.688 Å for NifEN and 1.209 Å for NifH), underscoring the high quality of the model. **c,** Structure of the AlF_4^--stabilized NifDK/NifH complex, with subunits, domains, and clusters shown as those in Fig. 1. The predicted NifEN/NifH complex model closely resembles the crystal structure of the AlF_4^--stabilized NifDK/NifH complex, particularly with respect to the arrangement of protein components and associated metalloclusters.

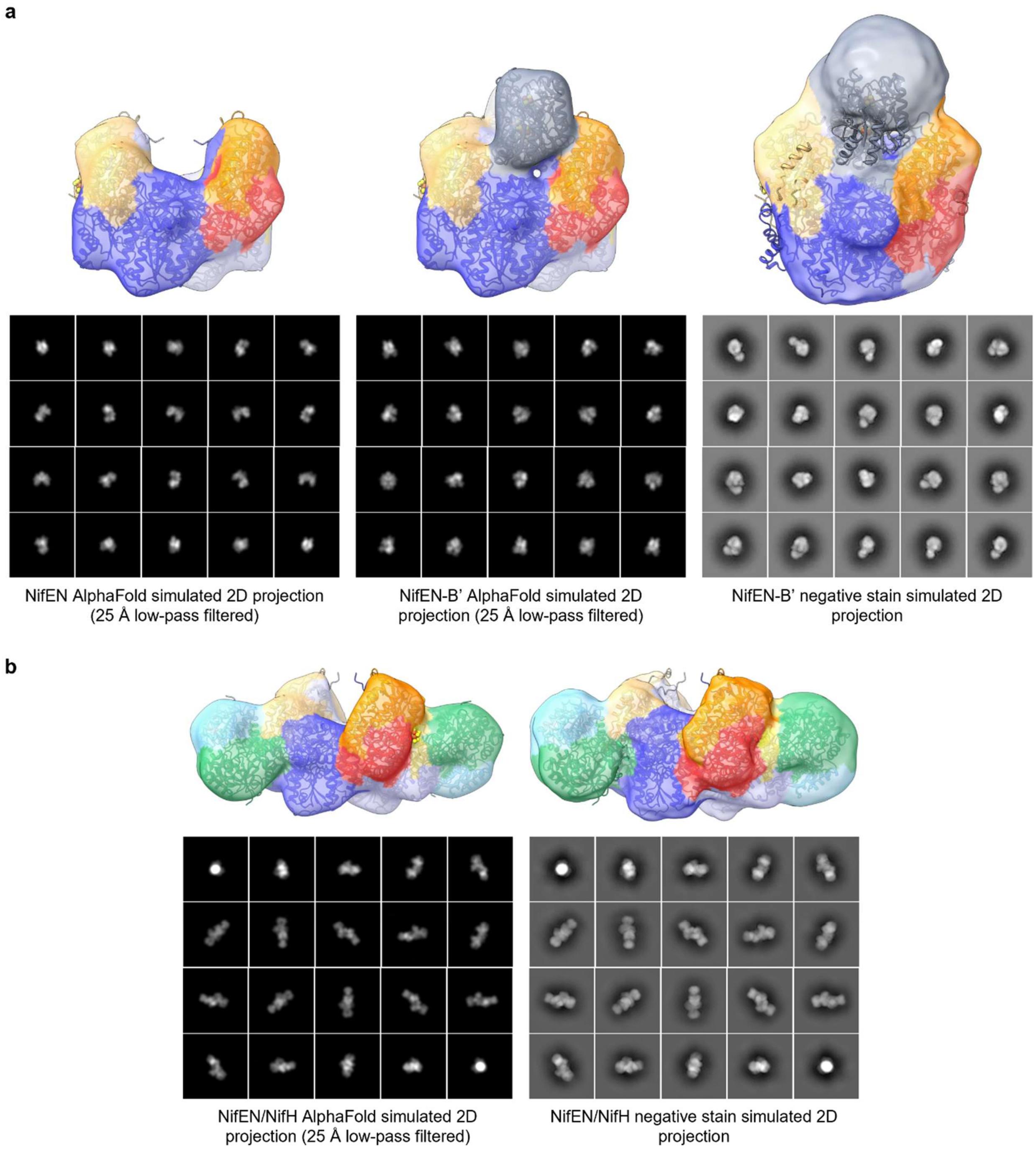

Supplementary Fig. 15 | Simulated 2D projections of the NifEN-B' fusion and the NifEN/NifH complex. a,b, Simulated 2D projections of the AlphaFold 3 models, lowpass filtered to 25 Å, and refined negative stain 3D reconstructions of (**a**) the NifEN-B' fusion and (**b**) the NifEN/NifH complex.

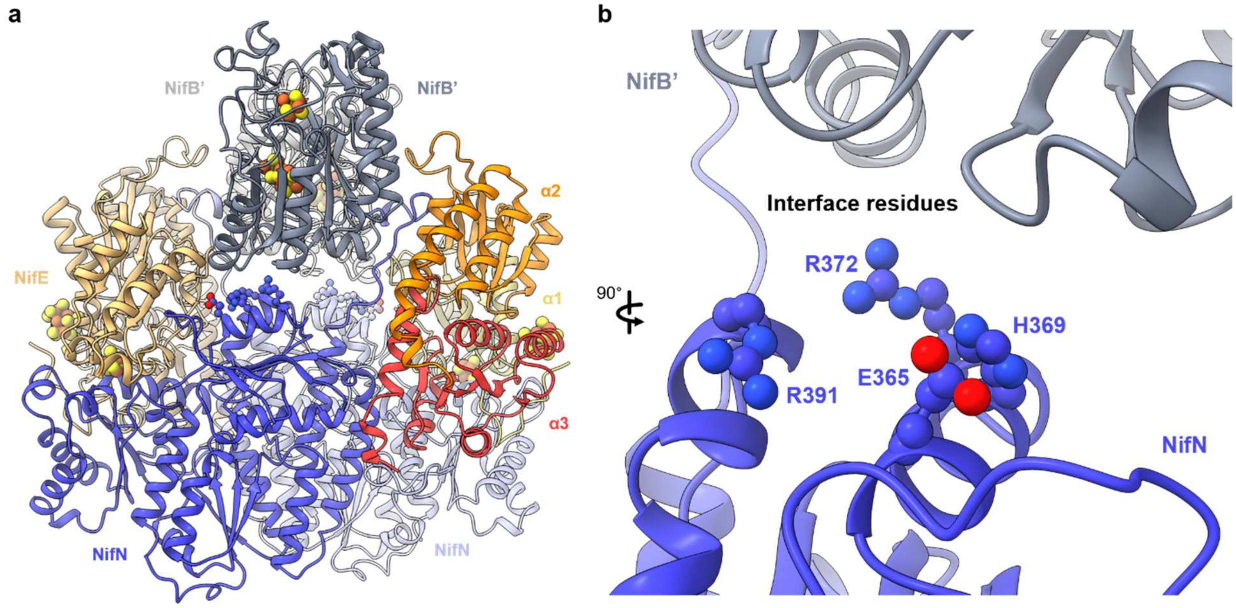

Supplementary Fig. 16 | The proposed interface residues between NifN and NifB'. a, The AlphaFold 3 model of NifEN-B' fusion with the proposed interface residues from NifN shown as ball-and-sticks. **b,** A closer view of the NifN-NifB' interface. The residues were chosen as described in the Methods.

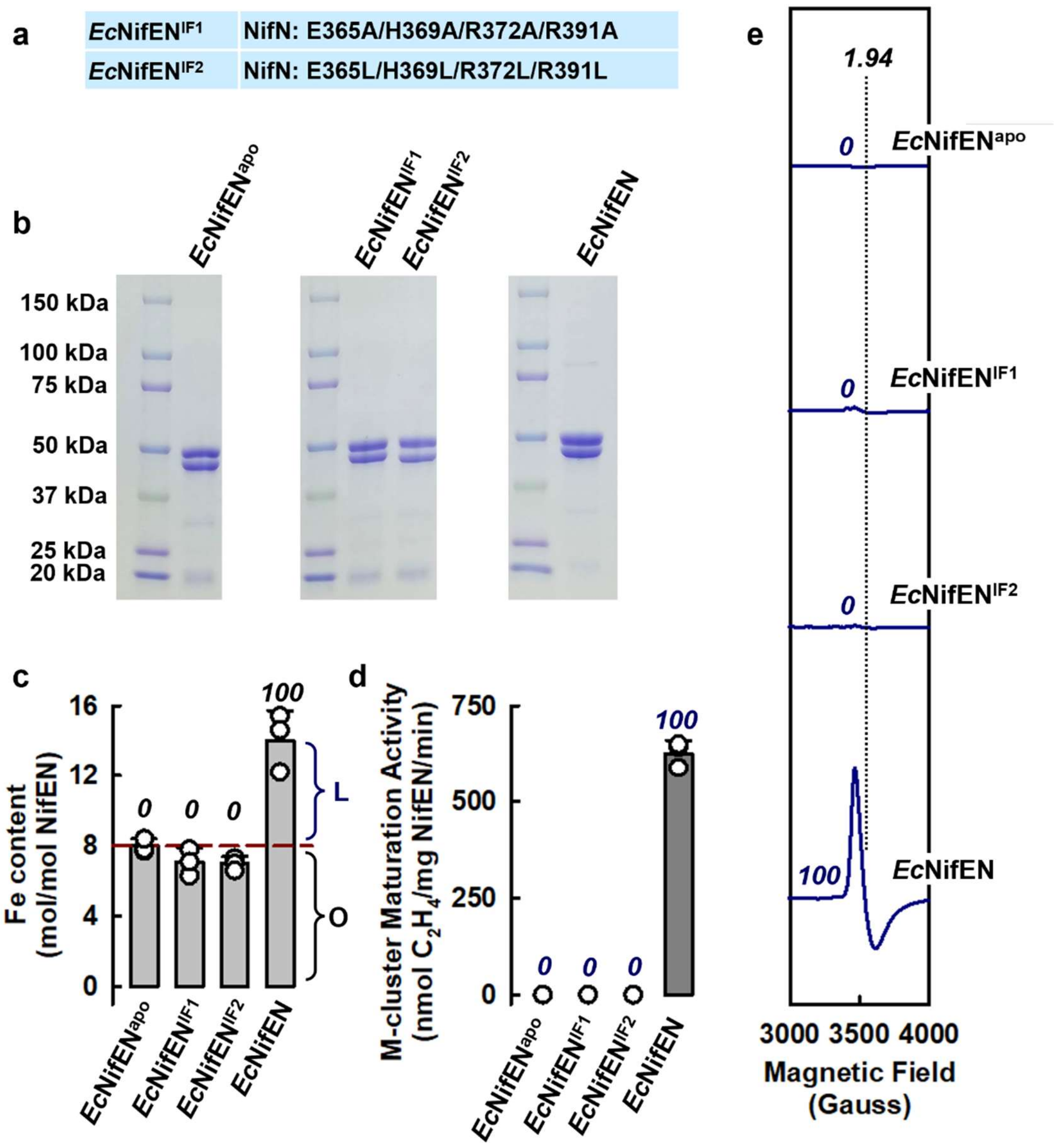

Supplementary Fig. 17 | Biochemical and spectroscopic characterization of NifEN variants at the interface between NifN and NifB'. a, Two NifEN variants containing mutations of four residues of NifN at the NifEN-NifB' interface (IF) were heterologously expressed in *E. coli*: *Ec*NifEN^{IF1}: E365A/H369A/R372A/R391A; *Ec*NifEN^{IF2}: E365L/H369L/R372L/R391L. **b-e,** SDS-PAGE (**b**), Fe content (n=3) (**c**), M-cluster maturation assay (n=3) (**d**), and EPR spectra (IDS-oxidized) (**e**) of the two NifEN variants along with the L-cluster deficient *Ec*NifEN^{apo} (expressed in the absence of NifB) and the L-cluster replete *Ec*NifEN (expressed in the presence of NifB). The L-cluster specific Fe content (**c**), M-cluster maturation activity (**d**), and intensity of the L-cluster specific EPR signal (**e**) of the two NifEN variants are shown in bold font and expressed as percentages, with the corresponding values of *Ec*NifEN set as 100%. The L-cluster specific Fe content was calculated by subtracting 8 Fe atoms in the two permanent O-clusters ([Fe_4S_4]) from the total Fe content (**c**). Data are presented as mean values ± SD (**c**, **d**).

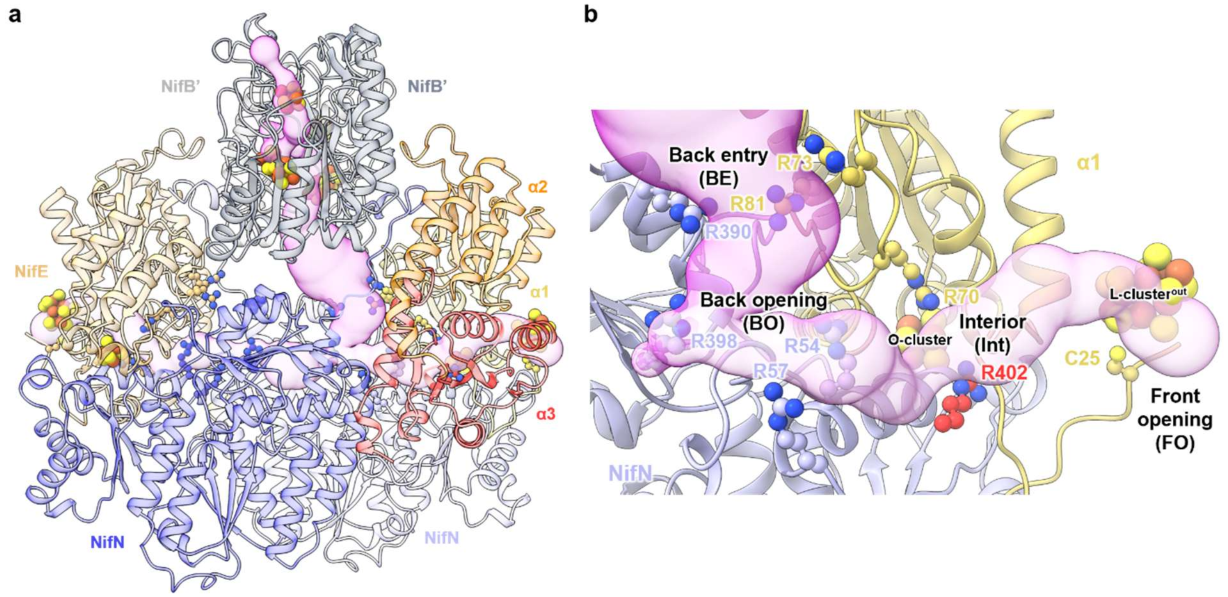

Supplementary Fig. 18 | The residues lining the proposed L-cluster trafficking tunnel. (**a**) The AlphaFold 3 model of NifEN-B' fusion with the residues potentially involved in L-cluster trafficking shown as ball-and-sticks. (**b**) A closer view of the proposed L-cluster trafficking tunnel. The residues located at the back entryway (BE), the back opening (BO), the interior (Int) and the front opening (FO) of the tunnel were chosen as described in the Methods.

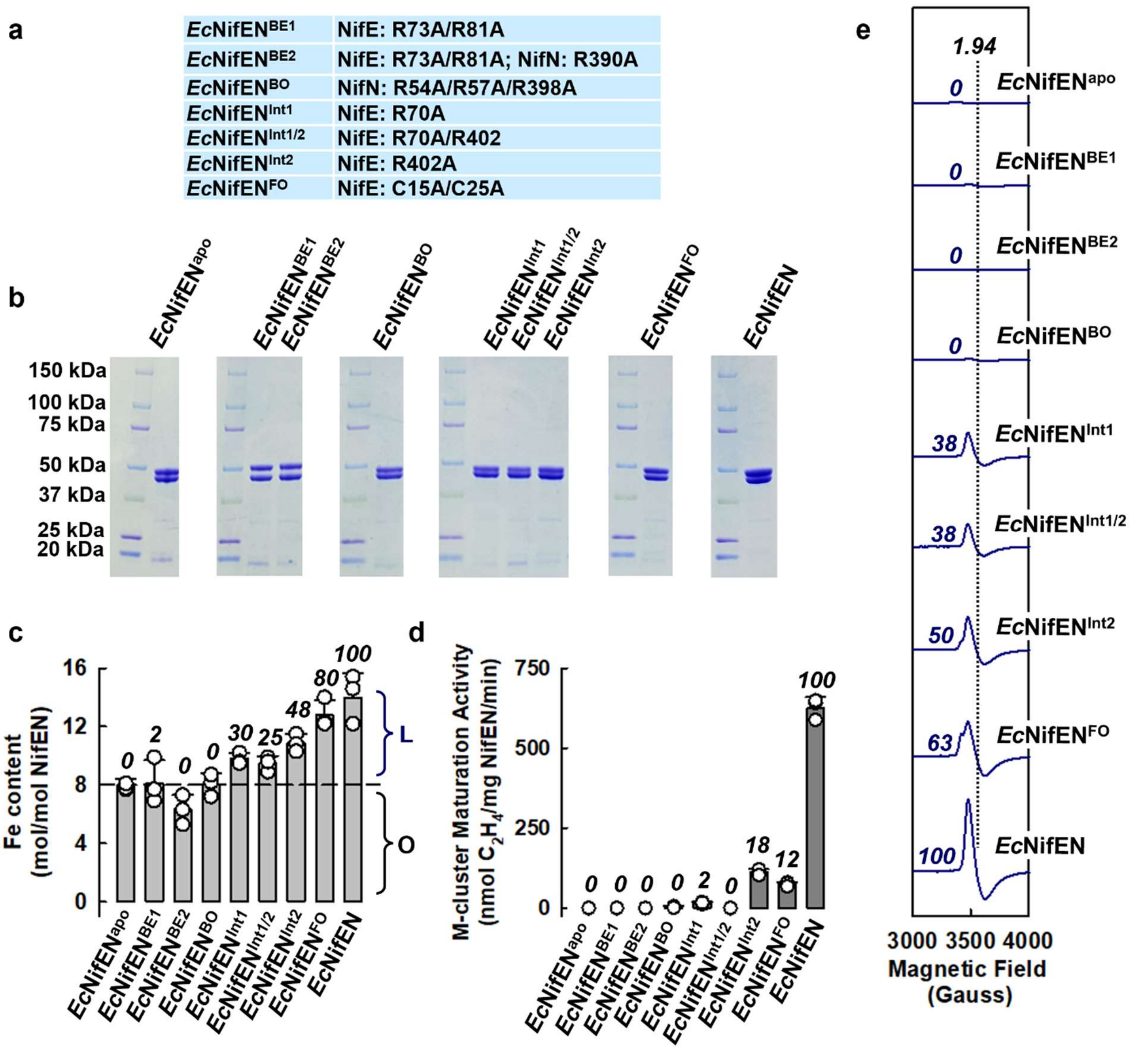

Supplementary Fig. 19 | Biochemical and spectroscopic characterization of NifEN variants targeting the proposed L-cluster trafficking tunnel. a, Seven NifEN variants containing mutations along the proposed L-cluster trafficking route, including those at the back entryway (BE), the back opening (BO), the interior (Int), and the front opening (FO) of the funnel, were heterologous expressed in *E. coli*: *Ec*NifEN$^{\mathrm{BE1}}$: R73A/R81A of NifE; *Ec*NifEN$^{\mathrm{BE2}}$: R73A/R81A of NifE and R390A of NifN; *Ec*NifEN^{BO}: R54A/R57A/R398A of NifN; *Ec*NifEN$^{\mathrm{Int1}}$: R70A of NifE; *Ec*NifEN$^{\mathrm{Int1/2}}$: R70A/R402A of NifEN; *Ec*NifEN$^{\mathrm{Int2}}$: R402A of NifE; *Ec*NifEN^{FO}: C15A/C25A of NifEN. **b-e,** SDS-PAGE (**b**), Fe content (n=3) (**c**), M-cluster maturation assay (n=3) (**d**), and EPR spectra (IDS-oxidized) (**e**) of the seven NifEN variants along with the L-cluster deficient *Ec*NifEN^{apo} (expressed in the absence of NifB) and the L-cluster replete *Ec*NifEN (expressed in the presence of NifB). The L-cluster specific Fe content (**c**), M-cluster maturation activity (**d**), and intensity of the L-cluster specific EPR signal (**e**) of the seven NifEN variants are shown in bold font and expressed as percentages, with the corresponding values of *Ec*NifEN set as 100%. Data are presented as mean values ± SD (**c**, **d**).

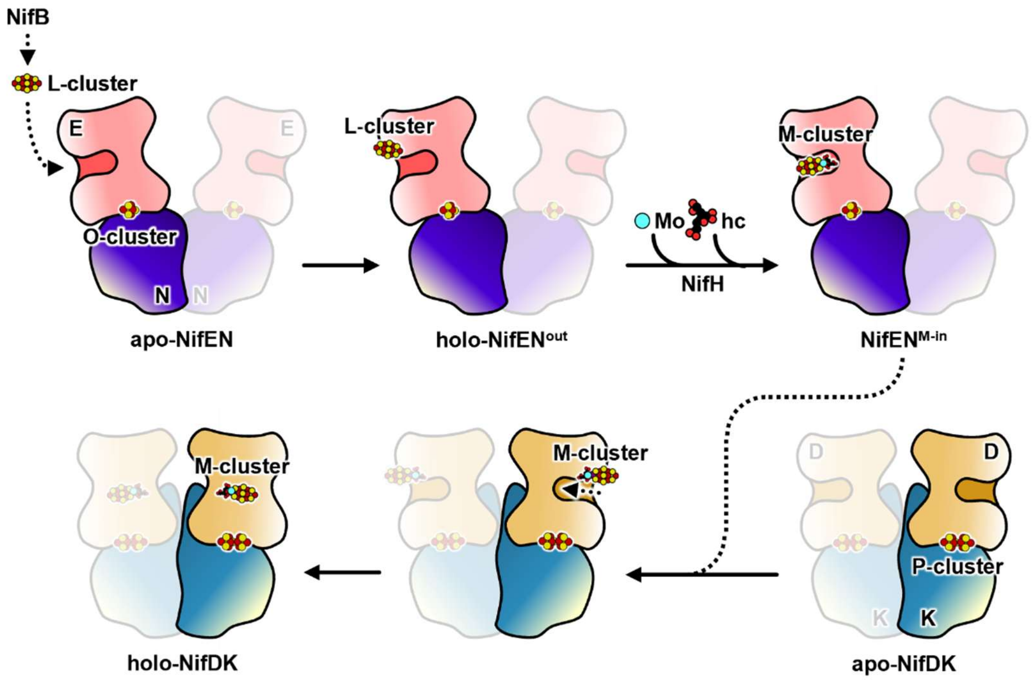

Supplementary Fig. 20 | Schematic presentation of the previously proposed biosynthetic events on NifEN and NifDK. The L-cluster is transferred from NifB to the entrance of the L-cluster insertion funnel on apo-NifEN, where it is positioned on the face opposite a valley formed by two NifE (α) subunits (as the slopes) and two NifN (β) subunits (as the base). This configuration gives rise to a surface-exposed L-cluster state, holo-NifEN^{out}. Subsequently, NifH accesses the L-cluster and inserts Mo and homocitrate (hc), generating a mature M-cluster that is then relocated to an interior binding site within NifEN, yielding NifEN$^{M\text{-}in}$. Upon docking with apo-NifDK, NifEN$^{M\text{-}in}$ releases the M-cluster from its interior site, allowing it to reposition on the surface of NifEN. The M-cluster is then transferred from NifEN to the surface of apo-NifDK and inserted through the cofactor insertion funnel into its final binding site, culminating in the formation of cluster-replete holo-NifDK.

Supplementary Tables

Supplementary Table 1 | Cryo-EM data collection and model statistics.

	Dataset 1	Dataset 2	Dataset 3
**Data collection**			
Microscope	Titan Krios		
Voltage (kV)	300		
Detector	Thermo Scientific Falcon 4i		
Pixel Size (Å)	0.743		
Total electron dose ($e^-/Å^2$)	60		
Defocus range (μm)	–1.0 to –2.0		
Energy filter slit width (eV)	6	10	6
# of movies	4,641	5,415	6,480

**Title**	**L-cluster free apo-NifEN expressed in E. coli**	**L-cluster inward bound holo-NifEN expressed in E. coli**
**Accession code**	PDB 9ONJ, EMD-70642	PDB 9ONK, EMD-70643
**Reconstruction**		
Software	CryoSPARC 4.2.1	
Symmetry	C1 (no symmetry)	
Selected movies	13,965 (1: 4,608; 2: 4,671; 3: 4,686)	
Final # of particles	126,644	40,670
Overall resolution (Å)	3.72	3.62
FSC threshold	0.143	
Map sharpening B factor ($Å^2$)	-154.08	-111.99
**Refinement**		
Model composition		
Non-hydrogen atoms	7,264	8,872
Protein residues	1,133	1,172
Ligands	0	2
R.m.s deviations		
Bond lengths (Å)	0.006	0.007
Bond angles (°)	0.834	0.886
Validation		
MolProbity score	1.67	1.44
Clashscore	6.20	4.01
Poor rotamers (%)	0.00	0.00
Ramachandran plot (%)		
Favored	95.24	96.20
Allowed	4.76	3.80
Outlier	0.00	0.00
