# Supplementary material for: Structural insights into metallocluster trafficking in the nitrogenase assembly scaffold NifEN
Source: Nat Catal. 2026 Mar 3;9(3):281–94. doi: 10.1038/s41929-026-01489-9 (PMC13031127; doi:10.1038/s41929-026-01489-9)
Supplement: Supplementary file 2 — Reporting Summary [file 41929_2026_1489_MOESM2_ESM.pdf]

nature portfolio

Corresponding author(s): Markus W. Ribbe, Shane Gonen, Yilin Hu

Last updated by author(s): 12/28/2025

Reporting Summary

Nature Portfolio wishes to improve the reproducibility of the work that we publish. This form provides structure for consistency and transparency in reporting. For further information on Nature Portfolio policies, see our Editorial Policies and the Editorial Policy Checklist.

Statistics

For all statistical analyses, confirm that the following items are present in the figure legend, table legend, main text, or Methods section.

n/a	Confirmed	
☐	☒	The exact sample size (*n*) for each experimental group/condition, given as a discrete number and unit of measurement
☒	☐	A statement on whether measurements were taken from distinct samples or whether the same sample was measured repeatedly
☒	☐	The statistical test(s) used AND whether they are one- or two-sided<br>*Only common tests should be described solely by name; describe more complex techniques in the Methods section.*
☒	☐	A description of all covariates tested
☒	☐	A description of any assumptions or corrections, such as tests of normality and adjustment for multiple comparisons
☐	☒	A full description of the statistical parameters including central tendency (e.g. means) or other basic estimates (e.g. regression coefficient) AND variation (e.g. standard deviation) or associated estimates of uncertainty (e.g. confidence intervals)
☒	☐	For null hypothesis testing, the test statistic (e.g. *F*, *t*, *r*) with confidence intervals, effect sizes, degrees of freedom and *P* value noted<br>*Give P values as exact values whenever suitable.*
☒	☐	For Bayesian analysis, information on the choice of priors and Markov chain Monte Carlo settings
☒	☐	For hierarchical and complex designs, identification of the appropriate level for tests and full reporting of outcomes
☒	☐	Estimates of effect sizes (e.g. Cohen's *d*, Pearson's *r*), indicating how they were calculated

Our web collection on statistics for biologists contains articles on many of the points above.

Software and code

Policy information about availability of computer code

Data collection	SerialEM
Data analysis	CryoSPARC 4.2.1; ChimeraX 1.9; PyMOL 2.5.2; Phenix 1.21.2-5419; Coot 0.9.8.95; MOLEonline 2.5; alphafoldserver.com; EasySpin 5.2.27

For manuscripts utilizing custom algorithms or software that are central to the research but not yet described in published literature, software must be made available to editors and reviewers. We strongly encourage code deposition in a community repository (e.g. GitHub). See the Nature Portfolio guidelines for submitting code & software for further information.

Data

Policy information about availability of data

All manuscripts must include a data availability statement. This statement should provide the following information, where applicable:

- Accession codes, unique identifiers, or web links for publicly available datasets
- A description of any restrictions on data availability
- For clinical datasets or third party data, please ensure that the statement adheres to our policy

All data is available from the authors upon reasonable request.
The cryo-EM maps associated with this study have been deposited to the Electron Microscopy Data Bank (EMDB) under accession codes EMD-70642 [https://www.ebi.ac.uk/pdbe/entry/emdb/EMD-70642] (L-cluster free apo-NifEN expressed in E. coli) and EMD-70643 [https://www.ebi.ac.uk/pdbe/entry/emdb/EMD-70643] (L-cluster inward bound holo-NifEN expressed in E. coli).

The atomic coordinates associated with this study have been deposited to the Protein Data Bank (PDB) under the accession codes 9ONJ [https://doi.org/10.2210/pdb9ONJ/pdb] (L-cluster free apo-NifEN expressed in E. coli) and 9ONK [https://doi.org/10.2210/pdb9ONK/pdb] (L-cluster inward bound holo-NifEN expressed in E. coli).
Previously reported models referred to in this manuscript can be found under accession codes 1L5H [https://doi.org/10.2210/pdb1L5H/pdb] (FeMo-cofactor Deficient Nitrogenase MoFe Protein), 1N2C [https://doi.org/10.2210/pdb1N2C/pdb] (Nitrogenase complex from Azotobacter vinelandii stabilized by ADP-tetrafluoroaluminate), 3PDI [https://doi.org/10.2210/pdb3PDI/pdb] (Precursor bound NifEN), 3U7Q [https://doi.org/10.2210/pdb3U7Q/pdb] (A. vinelandii nitrogenase MoFe protein at atomic resolution) and 7JMB [https://doi.org/10.2210/pdb7JMB/pdb] (Crystal structure of Nitrogenase iron-molybdenum cofactor biosynthesis enzyme NifB from Methanothermobacter thermautotrophicus with three Fe4S4 clusters).

Research involving human participants, their data, or biological material

Policy information about studies with human participants or human data. See also policy information about sex, gender (identity/presentation), and sexual orientation and race, ethnicity and racism.

Reporting on sex and gender	N/A
Reporting on race, ethnicity, or other socially relevant groupings	N/A
Population characteristics	N/A
Recruitment	N/A
Ethics oversight	N/A

Note that full information on the approval of the study protocol must also be provided in the manuscript.

Field-specific reporting

Please select the one below that is the best fit for your research. If you are not sure, read the appropriate sections before making your selection.

☒ Life sciences ☐ Behavioural & social sciences ☐ Ecological, evolutionary & environmental sciences

For a reference copy of the document with all sections, see nature.com/documents/nr-reporting-summary-flat.pdf

Life sciences study design

All studies must disclose on these points even when the disclosure is negative.

Sample size	N/A
Data exclusions	No data presented herein were excluded.
Replication	Supplementary Fig. 1: (a) M-cluster maturation assays and (b) Fe analyses were each performed on three independent samples (n=3). Shown in the figure are mean values +/- SD.<br>Supplementary Fig. 19: (c) Fe analyses and (d) M-cluster maturation assays were each performed on three independent samples (n=3). Shown in the figure are mean values +/- SD.<br>Supplementary Fig. 21: (c) Fe analyses and (d) M-cluster maturation assays were each performed on three independent samples (n=3). Shown in the figure are mean values +/- SD.
Randomization	N/A
Blinding	N/A

Reporting for specific materials, systems and methods

We require information from authors about some types of materials, experimental systems and methods used in many studies. Here, indicate whether each material, system or method listed is relevant to your study. If you are not sure if a list item applies to your research, read the appropriate section before selecting a response.

Materials & experimental systems

n/a	Involved in the study
☒	☐ Antibodies
☒	☐ Eukaryotic cell lines
☒	☐ Palaeontology and archaeology
☒	☐ Animals and other organisms
☒	☐ Clinical data
☒	☐ Dual use research of concern
☒	☐ Plants

Methods

n/a	Involved in the study
☒	☐ ChIP-seq
☒	☐ Flow cytometry
☒	☐ MRI-based neuroimaging

Plants

Seed stocks	N/A
Novel plant genotypes	N/A
Authentication	N/A
